# Physics-Informed Anomaly Detection and Fault Localization for Wind Turbines Using Multi-Strategy DASVDD and Bidirectionally Adjusted Reconstruction Error

**DOI:** 10.3390/s26216688

**Published:** 2026-10-22

**Authors:** Chen Zhang, Lijian Luo, Tianxiao Zhang

**Affiliations:** School of Electrical and Electronic Engineering, Hubei University of Technology, Wuhan 430068, China; 102410301@hbut.edu.cn (L.L.); 102500239@hbut.edu.cn (T.Z.)

**Keywords:** wind turbines, physics-informed, MS-DASVDD, anomaly detection, fault localization

## Abstract

Anomaly detection and fault localization are essential for the safe and economic operation of wind turbines and the mitigation of unplanned outages. A novel physics-informed anomaly detection and fault localization method is developed to address high false alarm rates and insufficient localization accuracy in small-sample scenarios. First, a physics-informed autoencoder, integrated with explicit algebraic physical constraints, is developed to verify the effective embedding of inherent physical relationships. Second, building upon this autoencoder, a multi-strategy deep autoencoder-based support vector data description (MS-DASVDD) for anomaly detection that incorporates physical constraint regularization, latent variable alignment, and transfer learning is developed, significantly reducing false alarms and missed detections with limited training data. Finally, a physics-informed method for bidirectional reconstruction error adjustment is proposed, which penalizes errors consistent with physical constraints while accentuating inconsistent ones, thus significantly boosting error-driven fault feature localization accuracy under limited fault samples. Case studies on two wind farms (40 MW and 30 MW) in Northern China show that the proposed physics-informed method markedly improves precision, recall, and F1 score, suppresses spiky anomaly scores, and enhances the contribution of fault features, confirming its effectiveness and robustness; sensitivity and comparative analyses further validate its superiority.

## 1. Introduction

**Figure 1:**
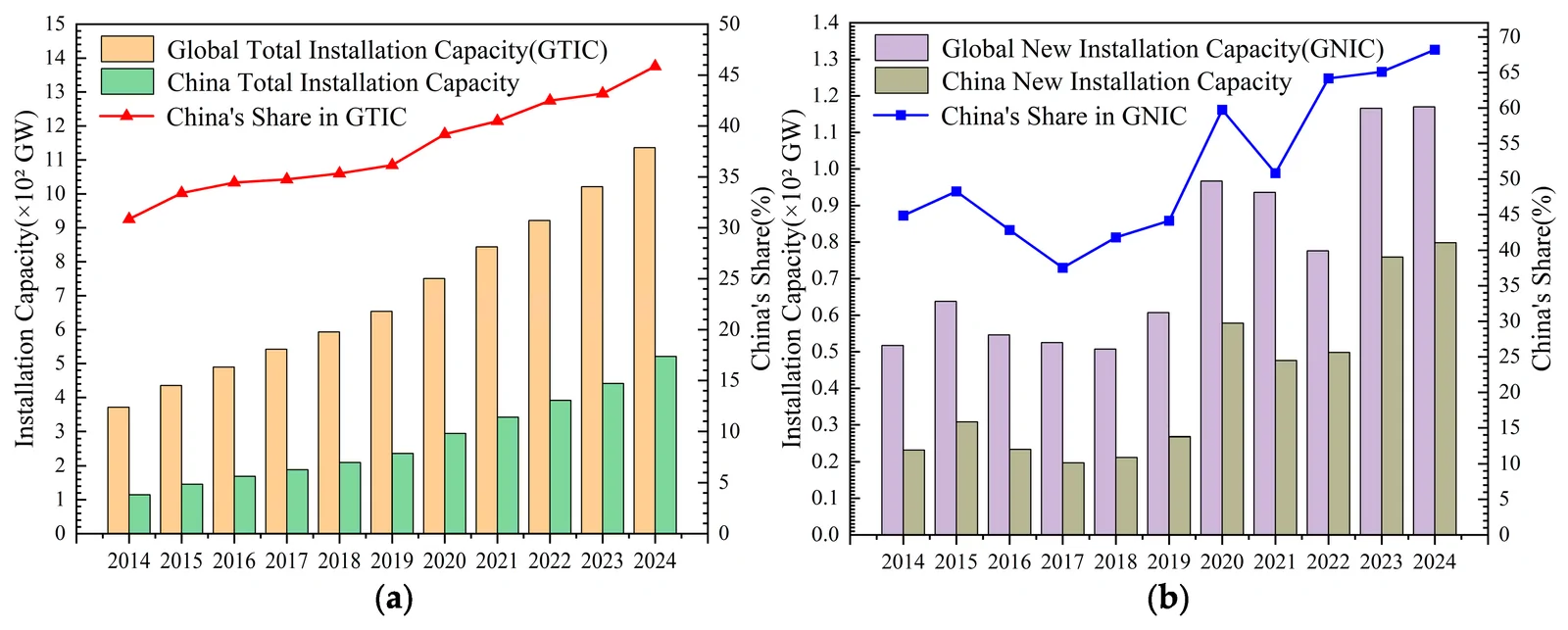
Wind energy installation capacity from 2014 to 2024: (**a**) Total installation capacity; (**b**) New installation capacity.

As a critical component of renewable energy, wind energy plays a vital role in the global energy transition. The Global Wind Energy Report reveals that the global newly installed wind power capacity hit a record high of 117 gigawatts (GW) in 2024, while the cumulative installed capacity reached 1136 GW [1]. As illustrated in Figure 1, wind power installed capacity in both the global and the Chinese markets exhibited a consistent upward trend from 2014 to 2024, reflecting a robust expansion of the market. According to the 2025 national power statistics released by the National Energy Administration of China, by the end of 2025, the cumulative installed capacity of wind power nationwide reached 640 million kW, while the newly added capacity was 119 million kW, representing a year-on-year growth of approximately 49% [2]. It is estimated that the cumulative installed capacity of wind power in China will reach 1.3 billion kW by 2030.

Nevertheless, the rapid expansion of installed wind power capacity has brought operational safety concerns to the forefront. Wind turbines are predominantly deployed in remote onshore regions or offshore environments, where they are continuously exposed to severe operational conditions, including long-term cyclic loading, extreme thermal variations, and salt-laden atmospheric corrosion. These environmental stressors contribute directly to the progressive deterioration of critical drivetrain components, such as gearboxes, generators, and main shaft bearings, thereby increasing the likelihood of unplanned downtime and catastrophic failure [3,4]. These degradation-induced failures not only lead to substantial losses in energy production and escalating operation and maintenance (O&M) expenditures, but may also precipitate severe safety incidents, including blade fractures and nacelle fires. Consequently, the development of robust early warning methodologies for incipient faults and anomalies in wind turbines and their key subsystems is of paramount importance. These approaches are essential for safeguarding operational stability, mitigating the risk of cascading failures, prolonging the service life of wind turbine assets, and reducing overall maintenance costs [5].

In recent years, machine learning-based data-driven modeling methods have been studied extensively and successfully applied to wind turbine modeling, monitoring, and diagnosis [6]. Existing research has shown that such data-driven models offer advantages in modeling accuracy and computational efficiency, yet they suffer from limited interpretability and generalization capability, making it difficult to simultaneously achieve high-precision anomaly detection and accurate fault localization [7,8]. It is therefore necessary to systematically review the development trajectory and technical limitations of wind turbine anomaly detection and fault localization methods.

Anomaly detection for wind turbines has undergone a paradigm shift from physics-based modeling to data-driven methodologies. Early-stage physics-based approaches, rooted in mechanistic modeling and finite element analysis, offer strong interpretability; however, the inherent nonlinearity, time-varying dynamics, and uncertainties render precise mathematical modeling exceedingly challenging. Signal processing techniques (spectral analysis, envelope demodulation, and wavelet transform) can extract fault-characteristic frequencies, yet fail to uncover complex interdependencies among high-dimensional multi-sensor measurements. Recently, deep learning architectures (deep autoencoders (DAE) [9], variational autoencoders (VAE) [10], generative adversarial networks (GAN) [11], and support vector data description (SVDD) [12]) have shown substantial promise in wind turbine anomaly detection by virtue of their exceptional nonlinear representation capacity. Nevertheless, purely data-driven approaches face two fundamental limitations. First, wind turbines generate power only within a limited wind speed band between the cut-in speed and the cut-out speed. Below the cut-in speed, the rotor remains stationary and no active operational data are recorded; above the cut-out speed, the turbine shuts down for protection, yielding almost no valid samples. Moreover, the transitional regimes near cut-in (where the pitch system first engages to regulate power) and near cut-out (where aerodynamic braking and emergency shutdown are initiated) occupy only a small fraction of the total operational time, resulting in a highly imbalanced Supervisory Control and Data Acquisition (SCADA) data distribution across wind speed regimes. Newly commissioned turbines face an even more severe situation due to their short operational history. When an anomaly detection model is trained on such skewed data, it tends to overfit the dominant operating regimes where the bulk of training samples reside, while the normal operating patterns in these under-represented cut-in, cut-out, and transitional regimes are insufficiently learned. This overfitting to the majority regimes causes the learned normal boundary to be overly narrow and poorly generalized to the sparse regimes. Consequently, normal fluctuations occurring in these under-represented regimes are misinterpreted as deviations from the learned normal boundary, producing elevated anomaly scores and triggering false alarms. Second, without physical prior guidance, purely data-driven models are prone to misinterpreting seasonal temperature drifts as thermal overload faults or erroneously correlating wind-speed fluctuations with drivetrain anomalies, yielding physically inconsistent diagnostic conclusions.

Crucially, these two challenges are interrelated: data sparsity constitutes an ill-posed inverse problem, whereas physical laws (conservation of energy, moment-of-momentum balance, and thermodynamic constraints) can serve as intrinsic regularizers that confine the hypothesis space and provide prior guidance in data-scarce regions [13]. This regularization rationale is directly embodied in physics-informed neural networks (PINNs), which incorporate physical laws into the loss function as residual terms, offering a viable solution to the above bottlenecks [14]. References [15,16] employed physics-informed neural networks (PINNs) for wind turbine applications, wherein residual terms derived from partial differential equations (PDEs) serve as the predominant vehicle for embedding physical knowledge, yielding competitive performance. However, current PINN-based physical constraint research remains heavily concentrated on the PDE-residual paradigm, with a notable absence of systematic modeling of the abundant explicit algebraic physical relationships (equalities, inequalities, monotonicity, and correlations) rooted in wind turbine engineering expertise, which constrains the generalizability and trustworthiness of physics-guided deep models for anomaly detection.

Fault localization in wind turbines constitutes a critical post-detection component of industrial anomaly detection, with its core objective centered on quantifying the contribution weights of massive SCADA-monitored variables to system anomaly deviation signatures [17]. The goal is to precisely identify key feature variables that trigger turbine faults from high-dimensional, strongly coupled, and nonlinear operational data, thereby furnishing fundamental evidence for fault tracing, defect discrimination, and preventive maintenance [18].

The prevailing methodologies can be categorized into three classes: mechanism-driven analytical modeling, data-driven multivariate statistical modeling, and emerging explainable artificial intelligence (XAI)-based diagnostic approaches. Mechanism-based analytical modeling represents the classical model-driven paradigm for fault localization. This approach constructs observation and parameter identification models grounded in the aerodynamic, mechanical, and electrical physics of wind turbines, and achieves precise fault localization through residual analysis between actual outputs and theoretical predictions [19]. Owing to its strong physical interpretability and minimal dependence on extensive training data, this method is well-suited for fault localization under simple operating conditions and steady-state component failures. Nevertheless, wind turbine systems exhibit pronounced nonlinearity and time-varying uncertainties, which render the construction of high-fidelity global mechanistic models exceedingly difficult, limiting their applicability to complex real-world scenarios. Data-driven multivariate statistical approaches exploit statistical regularities inherent in normal operational data. Techniques such as principal component analysis (PCA) and its kernelized extensions establish low-dimensional latent representations and assess sample deviations via squared prediction error (SPE), Hotelling’s T^2^, or reconstruction error [20]. Following anomaly detection, contribution plots and reconstruction-based contribution (RBC) methods pinpoint the dominant contributing variables [21]. However, these methods inherently presuppose that process data conform to a linear subspace or fixed-kernel implicit feature space, and exhibit pronounced sensitivity to outliers and missing data. When confronted with time-varying characteristics, nonlinear interactions, and noise perturbations pervasive in industrial processes, their monitoring performance deteriorates substantially, with false alarm rates inadequate for engineering requirements. XAI-based methods, built upon high-capacity models (e.g., deep neural networks and ensemble trees) that directly learn the input-output nonlinear mappings, leverage post hoc explanation techniques (SHapley Additive exPlanations (SHAP), Local Interpretable Model-agnostic Explanations (LIME), and permutation feature importance) to quantify feature contributions at both individual-sample and global levels, enabling black-box models to ascribe predictions to critical process variables with quantifiable interpretability [22,23]. References [24,25,26] respectively validate the effectiveness of data-driven methodologies for wind turbine fault localization along three technical routes, namely canonical variate analysis coupled with reconstruction-based contribution, principal component analysis with a relative variation contribution strategy, and SHAP-based explainable machine learning.

Collectively, these studies demonstrate that the localization performance of such approaches is critically governed by the availability of sufficient and representative training samples to ensure robust extraction of statistical regularities. However, in data-sparse regions (cut-in/cut-out wind speed regimes and early-stage operation of newly commissioned turbines), insufficient training samples render multivariate statistical and black-box models highly prone to overfitting, causing contribution plots or SHAP values to generate spurious variable attributions. Furthermore, being purely data-driven and devoid of physical priors, these approaches may misinterpret ambient temperature drift and wind speed fluctuations as critical fault variables, yielding physically inconsistent localization outcomes.

Therefore, this study is motivated by the need to reduce false alarms and improve fault-localization accuracy in small-sample wind-turbine scenarios via a novel physics-informed approach. First, a physics-informed autoencoder, integrated with explicit algebraic physical constraints, is developed to verify the effective embedding of inherent physical relationships. Second, building upon this autoencoder, an MS-DASVDD for anomaly detection that incorporates physical constraint regularization, latent variable alignment, and transfer learning is developed, significantly reducing false alarms and missed detections under insufficient training samples. Finally, a physics-informed method for bidirectional reconstruction error adjustment is proposed, which penalizes errors consistent with physical constraints while accentuating inconsistent ones, thus significantly boosting error-driven fault feature localization accuracy under limited fault samples.

The main contributions of this study include:

(1) A physics-informed autoencoder incorporating explicit algebraic physical relationships is used to validate their effective embedding.
(2) An MS-DASVDD-based anomaly detection method is developed, incorporating physical constraint regularization, latent variable alignment, and transfer learning, to reduce false alarms and missed detections.
(3) A physics-informed bidirectional reconstruction error adjustment method is proposed to penalize consistent errors and accentuate inconsistent ones, boosting fault localization accuracy.

## 2. Methodology

**Figure 2:**
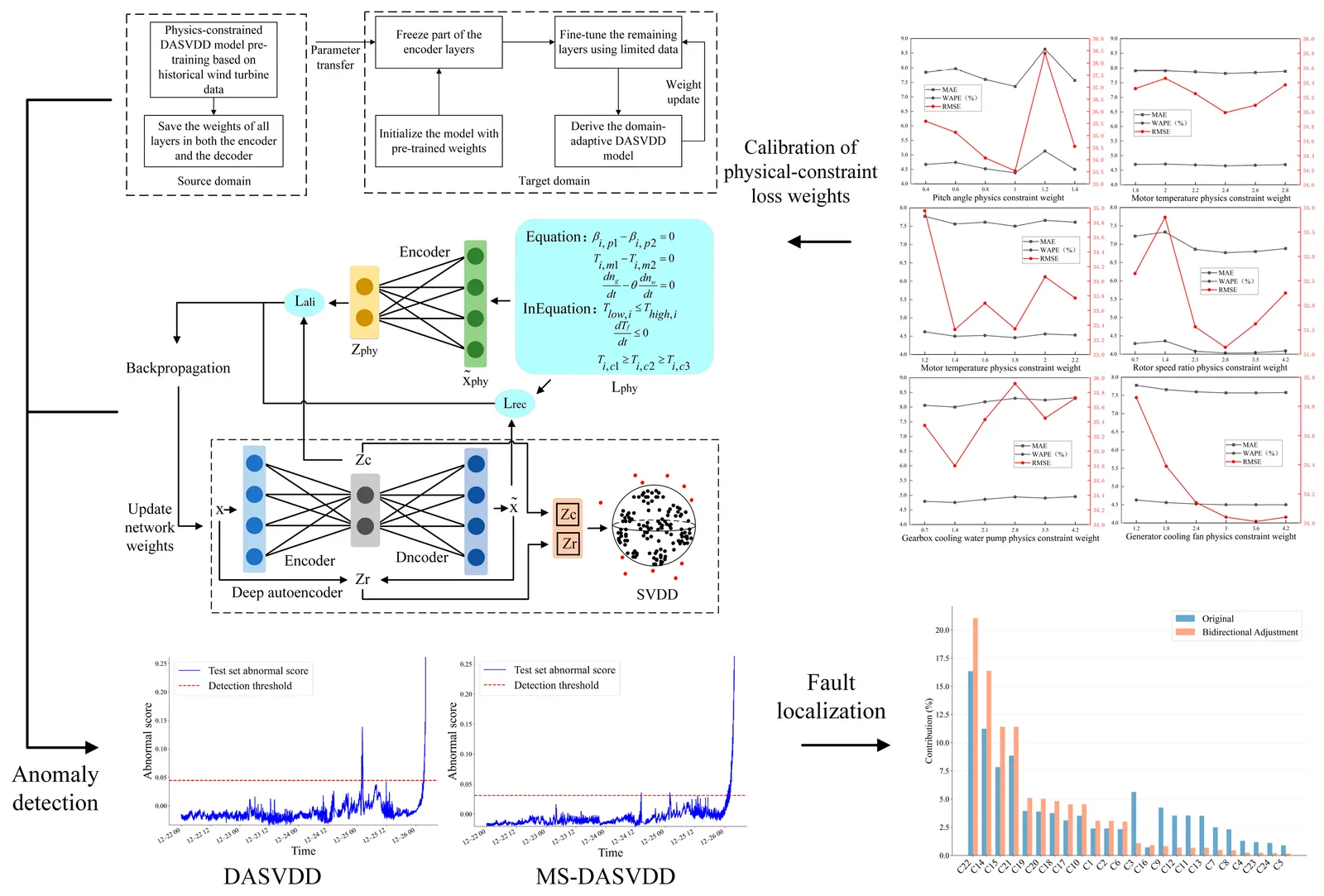
Physics-informed framework for anomaly detection and fault localization.

The framework of the proposed physics-informed anomaly detection and fault localization method for wind turbines is illustrated in Figure 2. Historical SCADA operational data of wind turbines are utilized to perform weight sensitivity analysis for autoencoder-based explicit algebraic physical constraints, so as to quantitatively calibrate the optimal weight parameters of each physical constraint. The calibrated optimal constraint weights are embedded into the DASVDD model to construct and train a physics-informed wind turbine DASVDD normal behavior model, and transfer learning strategies are further introduced to optimize model performance. On this basis, kernel density estimation is conducted on the model-generated anomaly scores to determine the anomaly detection threshold. Subsequently, SCADA data are fed into the fully trained physics-informed MS-DASVDD model to calculate anomaly scores and accurately identify abnormal turbine states. For the anomalous samples detected by the model, a physics-informed method for bidirectionally adjusting reconstruction errors is adopted for in-depth fault localization analysis, achieving accurate discrimination of wind turbine fault types.

### 2.1. Data from SCADA Systems

#### 2.1.1. Data Description

SCADA data were collected from two wind farms with installed capacities of 40 MW and 30 MW, respectively. The two wind farms are equipped with W2500-135 wind turbines (rated power of 2500 kW, rotor diameter of 135 m, and swept area of 14307 m^2^) and W2500-135 wind turbines (rated power of 2500 kW, rotor diameter of 135 m, and swept area of 14314 m^2^), respectively. The raw SCADA system records over 250 channels of signals. To construct a compact and physically interpretable monitoring set, variables were selected based on domain expert knowledge: signals directly reflecting the operating conditions of critical components and subsystems—including bearing temperatures, gearbox temperatures, generator stator and bearing temperatures, pitch system variables, rotational speeds, active power, and environmental parameters—were retained, while administrative and commissioning signals (e.g., communication status, remote/local control switches, and test mode flags) that carry no health-related information were excluded. No further statistical correlation-based filtering was applied, as the retained variables were deliberately chosen to cover all monitored subsystems with minimal redundancy. After selection, the same set of 26 variables with a sampling interval of 1 min was retained for condition monitoring, comprising 24 continuous variables and 2 discrete control variables. The two discrete control variables (gearbox cooling water pump status and generator cooling fan status) do not directly participate in model training but serve as auxiliary signals for activating the corresponding physical constraint losses, as listed in Table 1.

#### 2.1.2. Mathematical Formulation of Wind Turbine Physical Relationships

To accurately characterize the causal mechanisms among variables and enable interpretable anomaly detection and fault localization in wind turbine physical constraint modeling, this paper adopts a physics-informed strategy that formalizes both quantitative relationships (e.g., energy, mass, and momentum conservation) and qualitative relationships (e.g., monotonicity and correlations between temperature and other operational variables) by integrating physical functional boundaries with actual SCADA data. Table 2 summarizes a complete set of eight physical relationships derived from wind turbine operating mechanisms. Within this set, six physical relationships are selected to construct the multi-level mathematical constraint framework comprising equalities, inequalities, monotonicity constraints, and correlations, and each selected relationship corresponds to one loss term in the proposed model. The remaining two physical relationships in Table 2 are retained to fully describe the physical background of the wind turbine system and are not incorporated into the loss formulation of the current model. This treatment lays a physical constraint foundation for the subsequent anomaly detection and fault localization tasks. Among them, equality constraints and inequality constraints are the two most fundamental forms of explicit algebraic physical constraints within this framework, which are elaborated in detail in the following subsections.

- Equality

In wind turbine physical constraint modeling, equality constraints describe the instantaneous coupling and static equilibrium among various physical quantities at each time instant. These constraints are classified into rigid algebraic constraints and engineering approximate equality constraints based on their mathematical forms and physical interpretations. Rigid algebraic constraints arise from geometric definitions or physical laws with explicit formulations (e.g., the gear ratio definition). Engineering approximate equality constraints capture relationships that are not strictly equal due to manufacturing tolerances, installation errors, sensor noise, and environmental variations, but remain approximately equal within engineering precision (e.g., pitch motor temperature consistency and pitch angle consistency). This subsection formalizes and mathematically represents typical equality constraints in wind turbines following the above classification.

The rotor system of a wind turbine, which comprises the hub and the blades, is the core component responsible for energy capture, converting wind energy into mechanical energy that drives the rotation of the generator. As shown in Table 2 (①), owing to engineering imperfections such as manufacturing and installation tolerances, aeroelastic deformation of the blades, and differences in the dynamic responses of the pitch control system, the three pitch angles fluctuate dynamically and can therefore only be approximately equal in numerical value rather than strictly identical. The relationship among the three pitch angles can be expressed as:

*β_i_*_,*p*1_ ≈ *β_i_*_,*p*2_ ≈ *β_i_*_,*p*3_ (1)

where *β_i_*_,*p*1_, *β_i_*_,*p*2_, and *β_i_*_,*p*3_ denote the pitch angles of the three blades, respectively.

Furthermore, the pitch motors of the pitch system serve as external actuators that regulate the blade pitch within the rotor system. As shown in Table 2 (③), although these motors are exposed to the same thermal environment and operate under consistent working loads inside the hub, individual differences in heat dissipation and sensor errors allow them to achieve only a dynamic approximation in value. The relationship among the three pitch motors can be expressed as:

*T_i_*_,*m*1_ ≈ *T_i_*_,*m*2_ ≈ *T_i_*_,*m*3_ (2)

where *T_i_*_,*m*1_, *T_i_*_,*m*2_, and *T_i_*_,*m*3_ denote the motor temperatures of pitch motors 1, 2, and 3, respectively.

The drivetrain system of a wind turbine transmits the low-speed mechanical power captured by the rotor to the high-speed shaft of the generator through the main shaft, gearbox, and coupling. As shown in Table 2 (④), the rotor speed and the generator speed within this transmission chain satisfy a rigid drivetrain ratio relationship:

(3) $$\theta =\frac{n_{i,g}}{n_{i,w}}$$

where *θ* denotes the gearbox transmission ratio, i.e., the speed step-up ratio from the low-speed rotor shaft (i.e., the wind turbine rotor) to the high-speed generator shaft, which is a dimensionless constant determined by the gearbox design; *n_i_*_,*w*_ and *n_i_*_,*g*_ denote the rotor speed (i.e., the wind turbine rotor speed) and the generator speed, respectively, both measured in revolutions per minute (rpm). Accordingly, the two satisfy:

(4) $$\frac{dn_{i,g}}{\mathrm{dt}}-\theta \frac{dn_{i,w}}{\mathrm{dt}}=0$$

- Inequality

Inequality constraints are employed to characterize both the numerical ordering relationships (i.e., magnitude relations) and dynamic evolution trends (i.e., monotonicity) among physical quantities in wind turbines. This subsection provides a formalized description from three perspectives: the ordering relationships of gearbox bearing temperatures, the monotonicity of generator bearing temperature variation rates, and the multi-level temperature ordering relationships within the nacelle.

In the drive train of a wind turbine, the temperatures of the low-speed and high-speed gearbox bearings are governed by the operating status of the gearbox cooling water pump, which is regulated by the control system. As shown in Table 2 (⑤), when the pump is switched on, the low-speed bearing temperature is expected to remain lower than the high-speed bearing temperature; when the pump is switched off, the high-speed bearing temperature is expected to be lower. The relationship between the two can be expressed as:

Once the gearbox cooling water pump is switched on, after a certain settling period:

*T_i_*_,*low*_ ≤ *T_i_*_,*high*_ (5)

Once the gearbox cooling water pump is switched off, after a certain settling period:

*T_i_*_,*low*_ ≥ *T_i_*_,*high*_ (6)

where *T_i_*_,*high*_ and *T_i_*_,*low*_ denote the high-speed bearing temperature and the low-speed bearing temperature, respectively.

The power generation system of a wind turbine is a core subsystem that comprises the generator, its grid-connected converter, the excitation system, and the cooling and lubrication devices, and converts the mechanical energy delivered by the drive train into electrical energy that conforms to grid requirements. The temperature of the bearing at the non-drive end of the generator is affected by the switching state of the generator cooling fan in the control system. As shown in Table 2 (⑦), the on/off status of the cooling fan governs the rate of change of the bearing temperature at the non-drive end: when the fan is switched on, the temperature is expected to decrease, whereas when it is switched off, the temperature should increase. The trend of the bearing temperature variation at the non-drive end is defined as:

(7) $$\frac{dT_{i,f}}{\mathrm{dt}}=T_{i+1}-T_{i}$$

After a delay of *D* steps in the segment where the generator cooling fan remains continuously switched on:

(8) $$\frac{dT_{i,f}}{\mathrm{dt}}\leq 0$$

After a delay of *D* steps in the segment where the generator cooling fan remains continuously switched off:

(9) $$\frac{dT_{i,f}}{\mathrm{dt}}\geq 0$$

where *T_i_*_,*f*_ denotes the temperature change at the non-drive end, and *T_i_* and *T_i_*_+1_ denote the bearing temperatures at the free end of the *i*-th and *i* + 1-th samples after a delay of *D* steps, respectively.

Finally, under the combined influence of the cooling and lubrication system and the law of energy conservation (heat spontaneously transfers from hotter objects to colder ones). As shown in Table 2 (⑧)**,** the temperatures inside and outside the nacelle are expected to satisfy the following ordering: the nacelle cabinet temperature is greater than or equal to the internal nacelle temperature, which is in turn greater than or equal to the ambient temperature. This relationship can be expressed as:

*T_i_*_,*c*1_ ≥ *T_i_*_,*c*2_ ≥ *T_i_*_,*c*3_ (10)

where *T_i_*_,*c*1_, *T_i_*_,*c*2_, and *T_i_*_,*c*3_ denote the cabinet temperature, the nacelle temperature, and the ambient temperature, respectively.

### 2.2. Physics-Informed Anomaly Detection for Small Samples

**Figure 3:**
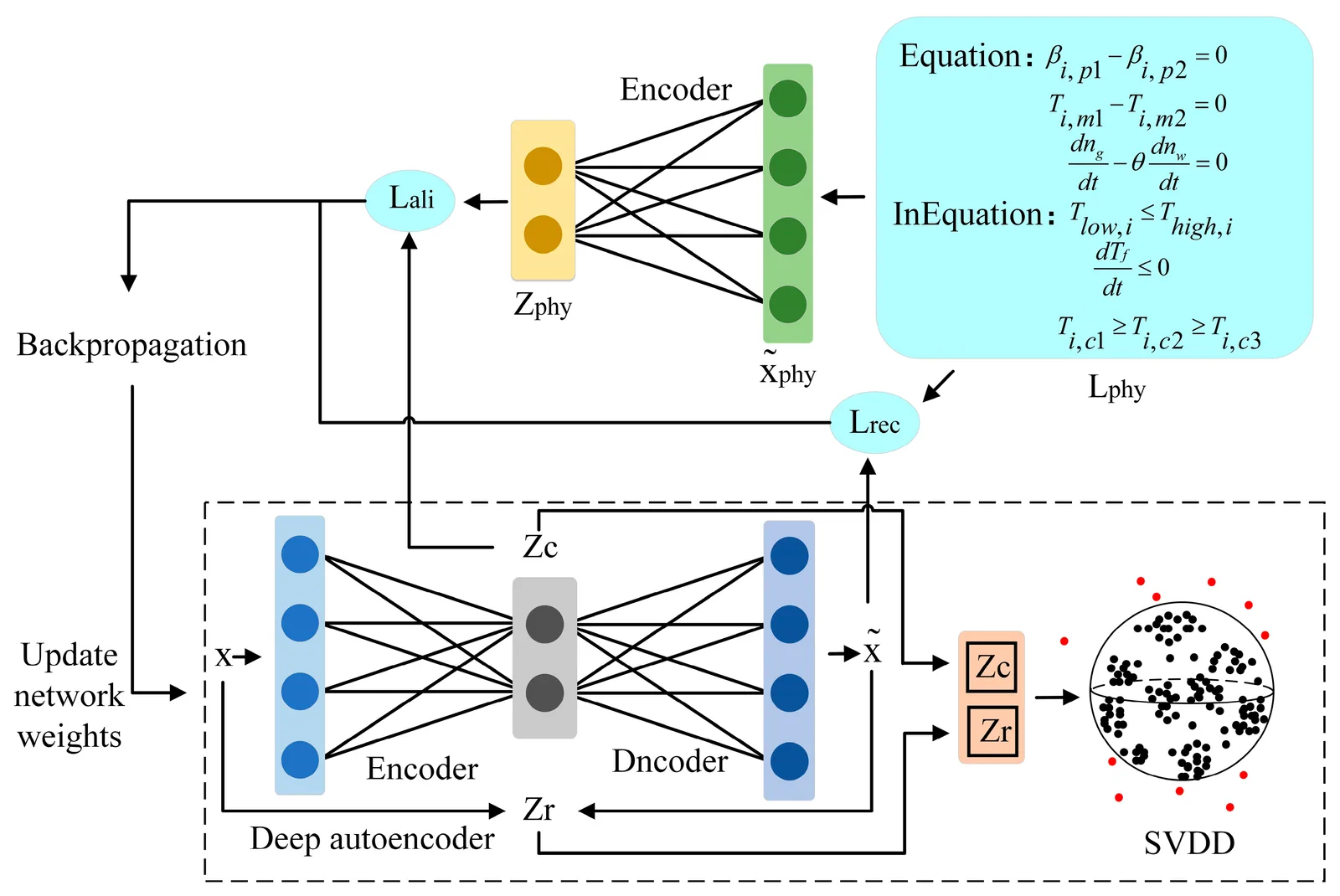
Structure of a physics-informed DASVDD.

The physics-informed DASVDD model adopts a collaborative architecture that combines reconstruction and discrimination, and its structure is shown in Figure 3. Specifically, raw SCADA data are first mapped by an autoencoder into a compact low-dimensional latent space, from which both latent features *Zc* and reconstruction features *Zr* are extracted. These two feature sets [*Zc*, *Zr*] are then concatenated along the channel dimension to form a joint feature vector that integrates global semantic information with local reconstruction deviations, and are subsequently fed into the SVDD module, where anomaly decisions are derived by minimizing the distances of normal samples to the center of the hypersphere. For physics knowledge embedding, the model implements a hierarchical constraint strategy consisting of direct and indirect constraints. Under the direct constraint, physical laws such as pitch angle consistency and the heat balance equation are formulated as equality or inequality constraints and are incorporated as residual penalty terms that regularize the reconstruction outputs of the autoencoder, thereby ensuring that the reconstructed results comply with the fundamental physical laws of wind turbines. Under the indirect constraint, the physically corrected reconstruction $\tilde{X}\mathrm{phy}$ outputs are re-projected into the latent space through a compression mapping that is structurally identical to the encoder and shares its parameters, yielding a latent representation guided by the embedded physics knowledge. The distributional discrepancy between this representation and the original latent variables produced by the autoencoder is then minimized by mean squared error, thereby transferring the physical knowledge into the latent representation in an indirect manner. Finally, the gradients of both the direct and indirect physical constraint terms [*Lrec*, *Lali*] jointly update all trainable parameters of the DASVDD framework through backpropagation, enabling the model to learn a compact decision boundary that is both physically consistent and discriminative in regions with sparse data.

#### 2.2.1. Mechanism Driven by Physical Constraints in the Autoencoder

To achieve physically consistent regularization for the reconstruction quality of the autoencoder under small-sample conditions, the model obtains reconstructed values in each forward pass and employs a physics residual computation module to extract the feature subset complying with the physical laws of wind turbines. Among the eight physical relationships summarized in Table 2, six are selected and constructed as physics residual terms to serve as regularization constraints. These six residual terms are directly imposed on the decoder’s output space to force the reconstructed outputs to follow wind-turbine physical priors, regulate the distribution of reconstructed values, and prevent the model from generating physically implausible reconstructions. The correspondence between the eight physical relationships in Table 2 and the six loss terms used in the proposed model is summarized in Table 3, which lists the physical relationships, applicable conditions, tolerance parameters, associated loss functions, and the corresponding index in Table 2.

The detailed mathematical formulations of the six physical constraints listed in Table 3 are presented below, following the same order as in the table.

Pitch angle constraint loss:

(11) $$\begin{array}{l} \beta_{i,p}=\max(0,\left|\beta_{i,p1}-\beta_{i,p2}\right|-\tau_{\mathrm{pitch}})+\max(0,\left|\beta_{i,p2}-\beta_{i,p3}\right|-\tau_{\mathrm{pitch}})+\\ \max(0,\left|\beta_{i,p3}-\beta_{i,p1}\right|-\tau_{\mathrm{pitch}}) \end{array}$$

(12) $$L_{\mathrm{pitch}}=\omega_{\mathrm{pitch}}\cdot \frac{1}{N}\sum_{i=1}^{N}\beta_{i,p}$$

where *ω_pitch_* and *τ_pitch_* denote the pitch angle constraint weight and the fluctuation tolerance of the pitch angle feature, respectively.

Rotor speed ratio constraint loss:

(13) $$L_{\mathrm{speed}}=\omega_{\mathrm{speed}}\cdot \frac{1}{N}\sum_{i=1}^{N}\left[\max\left(\left|n_{i,g}-\theta \cdot n_{i,w}\right|-\theta \cdot n_{i,w}\cdot \tau_{\mathrm{speed}}\right)\right]$$

where *ω_speed_* and *τ_speed_* denote the rotor speed ratio constraint weight and the relative tolerance, respectively.

Nacelle temperature ordering constraint loss:

*T_i_*_,*c*_ = max(0,−(*T_i_*_,*c*1_ − *T_i_*_,*c*2_ + *τ_cba_*)) + max(0,−(*T_i_*_,*c*2_ − *T_i_*_,*c*3_ + *τ_cba_*)) (14)

(15) $$L_{\mathrm{cba}}=\omega_{\mathrm{cba}}\cdot \frac{1}{N}\sum_{i=1}^{N}T_{i,c}$$

where *ω_cba_* and *τ_cba_* denote the nacelle temperature ordering constraint weight and the fluctuation tolerance of the temperature feature, respectively.

Pitch motor temperature constraint loss:

(16) $$\begin{array}{l} T_{i,m}=\max(0,\left|T_{i,m1}-T_{i,m2}\right|-\tau_{\mathrm{motor}})+\max(0,\left|T_{i,m2}-T_{i,m3}\right|-\tau_{\mathrm{motor}})+\\ \max(0,\left|T_{i,m3}-T_{i,m1}\right|-\tau_{\mathrm{motor}}) \end{array}$$

(17) $$L_{\mathrm{motor}}=\omega_{\mathrm{motor}}\cdot \frac{1}{N}\sum_{i=1}^{N}T_{i,m}$$

where *ω_motor_* and *τ_motor_* denote the pitch motor temperature constraint weight and the fluctuation tolerance of the motor temperature feature, respectively.

Gearbox cooling pump constraint loss:

Thermal inertia may temporarily reverse the temperature ordering between the low-speed and high-speed gearbox bearings during the activation/deactivation transients of the cooling pump; therefore, this constraint is applied only to steady operating segments under normal operating conditions and is encoded as a hinge loss, which softly penalizes any violation beyond a tolerance rather than enforcing it as a hard rule.

Let *M_on_* and *M_off_* denote the sets of samples with the cooling pump switched on and off, respectively, and let *τ*_1_ be the temperature tolerance. For pump-on samples, the high-speed bearing is expected to be warmer than the low-speed bearing, and the violation of sample *i* is measured by *e_i_* = max(0, *T_i_*_,*low*_ − *T_i_*_,*high*_ − *τ*_1_). For pump-off samples, the expected ordering is reversed, giving *e_i_* = max(0, *T_i_*_,*high*_ − *T_i_*_,*low*_ − *τ*_1_). Denoting by *η_on_* and *η_off_* the expected consistency levels, the stage losses are:

(18) $$L_{\mathrm{on}}=\max\left(0,\eta_{\mathrm{on}}-\left(1-\frac{\sum_{i\in M_{\mathrm{on}}}e_{i}}{\left|M_{\mathrm{on}}\right|}\right)\right)$$

(19) $$L_{\mathrm{off}}=\max\left(0,\eta_{\mathrm{off}}-\left(1-\frac{\sum_{i\in M_{\mathrm{off}}}e_{i}}{\left|M_{\mathrm{off}}\right|}\right)\right)$$

The total loss is defined as:

*L_pump_* = *ω_pump_* · (*L_off_* + *L_on_*) (20)

where *ω_pump_* denote the pump constraint weight.

Generator cooling fan constraint loss:

The same thermal-lag mechanism is used for the generator cooling fan. Let *T_i_* be the generator bearing temperature at the non-drive end, *f_i_* ∈ {0, 1} denote the fan state, and Δ*T_i_* = *T_i_* − *T_i_*_+1_ denote the temperature difference between adjacent samples. A maximal segment *S* = [*start*, *end*) with a constant fan state is retained only when its length exceeds a minimum duration *N*_min_. The first *D* samples after each switching event are excluded to avoid the invalid transition interval near the thermal-lag boundary. For a segment in which the fan remains continuously switched on, the steady-state samples are expected to satisfy Δ*T_i_* ≥ *τ*_2_, and the per-sample violation is *v_i_* = max(0, *τ*_2_−Δ*T_i_*). For a segment in which the fan remains continuously switched off, heat generation should prevent further cooling; therefore, only a continued decrease beyond the tolerance is penalized, i.e., *v_i_* = max(0, Δ*T_i_* − *τ*_2_). Summing over the steady response indices *i* = *start* + *D*, …, *end* − 2 yields:

(21) $$L_{\mathrm{on},\mathrm{seg}}=\sum_{i=\mathrm{start}+D}^{\mathrm{end}-2}\max\left(0,\tau_{2}-\Delta T_{i}\right)$$

(22) $$L_{\mathrm{off},\mathrm{seg}}=\sum_{i=\mathrm{start}+D}^{\mathrm{end}-2}\max\left(0,\Delta T_{i}-\tau_{2}\right)$$

The total loss is defined as:

(23) $$L_{\mathrm{fan}}=\omega_{\mathrm{fan}}\cdot \min\left(\frac{\sum_{\mathrm{seg}}L_{\mathrm{on},\mathrm{seg}}+L_{\mathrm{off},\mathrm{seg}}}{\sum_{\mathrm{seg}}N_{\mathrm{seg}}\cdot \omega_{\mathrm{seg}}\cdot \Delta T_{\max}},1\right)$$

where *D* denotes the number of delay steps, with the first *D* samples excluded from the loss computation; *N_seg_* denotes the number of sample pairs within a segment; *ω_seg_* denotes the segment weight, which is set identically for the on and off states; Δ*T*_max_ denotes the bearing temperature range at the non-drive end; *τ*_2_ denotes the temperature tolerance; and *ω_fan_* denotes the fan constraint weight.

The total physics constraint loss is defined as:

(24) $$L_{\mathrm{phys}}^{\mathrm{direct}}=L_{\mathrm{pitch}}+L_{\mathrm{speed}}+L_{\mathrm{cba}}+L_{\mathrm{motor}}+L_{\mathrm{pump}}+L_{\mathrm{fan}}$$

Although the above direct constraints ensure the physical plausibility of the reconstructed values and filter out the influence of physically inconsistent samples at the output, the physics knowledge acts only on the output of the decoder and is not effectively transferred to the latent representation extracted by the encoder. In regions with sparse data, the latent variables, lacking sufficient supervision, may deviate from a physically consistent feature distribution; meanwhile, noisy samples in the original input that violate physical relationships can still interfere with the latent feature extraction of the encoder, thereby degrading the discriminative performance of the subsequent SVDD decision layer. To address this issue, this paper proposes a latent alignment constraint that propagates the physics knowledge backward from the reconstruction output to the latent space.

Let *f_θ_*(⋅) denote the encoding mapping of the original autoencoder applied to an input sample *x*, and *g_ϕ_*(⋅) denote the corresponding decoding mapping; the original latent variable and the reconstructed value are then given by *Z_c_* = *f_θ_*(⋅) and $\hat{x}=g_{\phi }\left(\cdot \right)$, respectively. Re-inputting the reconstruction $\hat{x}$, which has been corrected by the direct physical constraints, into the encoder yields the physics-guided latent variable:

(25) $$Z_{\mathrm{phys}}=f_{\theta }\left(\hat{x}\right)$$

Since the mapping *f_θ_*(⋅) shares identical parameters in the two mappings, *Z_c_* and *Z_phys_* lie in the same feature space and can therefore be compared directly. The latent alignment loss is defined as:

(26) $$L_{\mathrm{align}}=\frac{1}{N}{\sum_{i=1}^{N}\left\|Z_{c}^{\left(i\right)}-Z_{\mathrm{phys}}^{\left(i\right)}\right\|}_{2}^{2}$$

This loss measures the distributional discrepancy between the original latent variable and the physics-guided latent variable by means of the mean squared error. By minimizing the alignment loss *L_align_*, the physics consistency knowledge is propagated backward from the reconstruction layer to the latent space through the encoder that shares its parameters, so that the latent variables not only preserve the distribution characteristics of the original data but also embed constraint information consistent with physical laws, effectively suppressing the influence of noisy samples that violate physical relationships in the original input on the feature distribution in the latent space. This mechanism addresses the core issue of insufficient supervision in the latent space under small-sample conditions and provides a more physically consistent and interference-robust compact feature distribution for the SVDD hypersphere.

#### 2.2.2. Support Vector Data Description

SVDD translates the one-class description problem into a hypersphere optimization problem. Within the single-class feature space spanned by the fused feature vectors, it seeks a hypersphere of minimum volume that encloses all normal samples, and introduces slack variables that allow a small number of samples to exceed the sphere boundary, thereby enhancing robustness against outliers and noise and yielding a compact data description characterized by the center and radius of the hypersphere. Specifically, let *Z_i_* ∈ *R^d^*(*i* = 1, 2, …, *N*) denote the fused feature vectors, *c* denote the center of the hypersphere, and *R* denote its radius. The model learns the hypersphere parameters by minimizing the following loss function:

(27) $$L_{\mathrm{SVDD}}=R^{2}+\frac{1}{\mathrm{\nu N}}\sum_{i=1}^{N}\max\left(0,{\left\|z_{i}-c\right\|}^{2}-R^{2}\right)$$

where *ν* is the parameter that controls the proportion of allowed outliers, and $\max\left(0,{\left\|z_{i}-c\right\|}^{2}-R^{2}\right)$ is the slack variable that allows a small number of abnormal samples to exceed the radius range. Under the condition of using the Radial Basis Function (RBF) kernel, the squared distance between the feature vector *z_i_* and the center *c* is computed by the kernel trick as:

(28) $${\left\|z_{i}-c\right\|}^{2}=2-2k\left(z_{i},c\right)$$

where $k\left(z_{i},c\right)=\exp\left(-\gamma \cdot {\left\|z_{i}-c\right\|}^{2}\right)$ is the RBF kernel similarity, and *γ* is the bandwidth parameter of the kernel. After training, the anomaly score of the sample *s_i_* is defined as:

(29) $$s_{i}={\left\|z_{i}-c\right\|}^{2}-R^{2}$$

A sample with *s_i_ > *0 lies outside the hypersphere and is identified as anomalous; otherwise, it is considered normal.

#### 2.2.3. Transfer Learning

**Figure 4:**
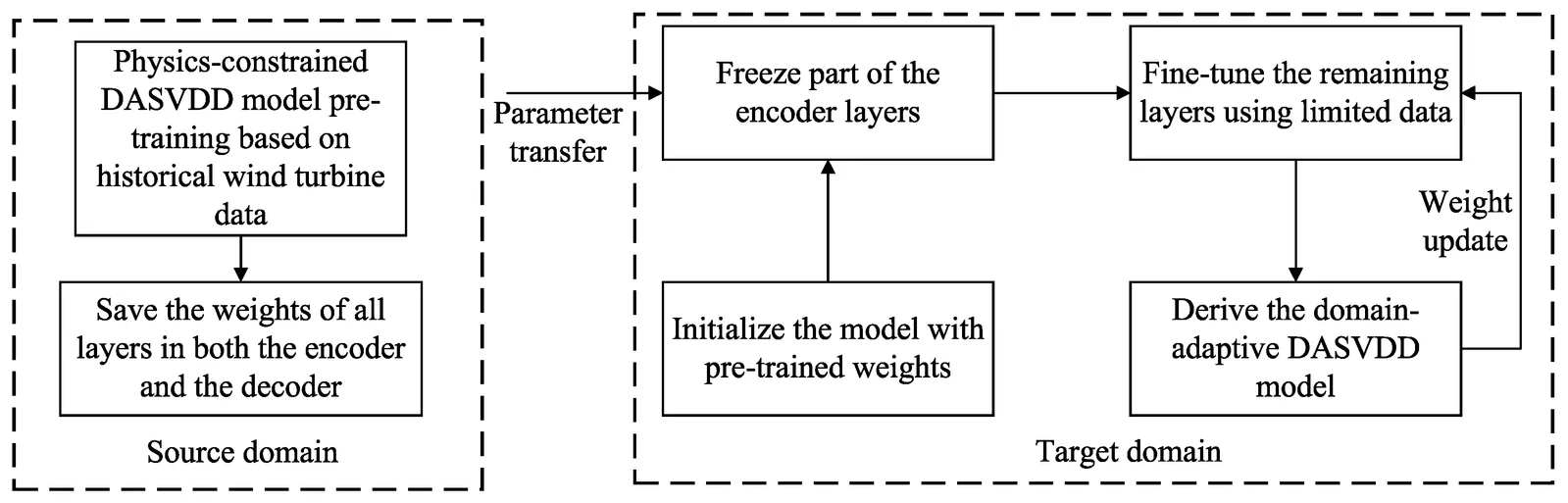
Flowchart of the transfer learning process.

Newly commissioned wind turbines only accumulate a small amount of data under normal operating conditions in the initial grid connection stage, which is insufficient to support the thorough training of deep autoencoders from scratch. Transfer learning provides an effective solution for model training with limited samples by transferring knowledge learned from source domain turbines with sufficient data to target domain turbines with data scarcity [27]. The core principle is that although individual differences exist between wind turbines in the source domain and target domain, both comply with the same fundamental physical laws, such as kinematic relations of drive trains and thermodynamic transfer paths. Therefore, encoder parameters pretrained on source domain data are capable of extracting general physical features of wind turbines. By transferring such knowledge to the target domain and conducting fine-tuning with a small volume of local data, the model can rapidly adapt to the operating characteristics of target turbines under limited sample conditions, thereby avoiding overfitting and generalization performance degradation caused by insufficient data. The corresponding flowchart of the transfer learning process is shown in Figure 4.

In the source domain pretraining stage, wind turbines with sufficient historical data from the same wind farm are selected as the source domain, and the DASVDD model is fully trained with the full normal operation data of these turbines. After the completion of pretraining, all network weights of the encoder and decoder are saved as knowledge carriers. In the target domain fine-tuning stage, the pretrained weights are loaded into the target domain model for newly commissioned turbines as initial parameters, and subsequent fine-tuning is performed using a small number of normal samples from the target domain. To balance the retention of general features and the adaptation of domain characteristics, the parameters of the first two encoder layers are frozen during fine-tuning, while the weights of the remaining layers are updated. This operation retains the general physical feature representations learned from the source domain and enables deep networks to make local adjustments specific to the target domain. Notably, the source domain and target domain adopt completely identical physical constraint types, residual calculation methods and regularization weight configurations throughout the entire transfer learning process. Specifically, turbines in the two domains share six types of physical constraints and the corresponding weight coefficients. This consistent design of physical constraints ensures the seamless transfer of physical prior knowledge among different turbines. It further guarantees that the physical rationality regularization mechanism learned in the pretraining stage is completely transferred to the fine-tuning stage, thereby providing continuous inductive bias for few-shot training in the target domain.

#### 2.2.4. Anomaly Detection

By integrating all the loss terms defined above, the total loss function of the model is formulated as:

(30) $$L_{\mathrm{total}}=\lambda_{1}L_{\mathrm{recon}}+L_{\mathrm{phys}}^{\mathrm{direct}}+\alpha L_{\mathrm{align}}+\lambda_{2}L_{\mathrm{svdd}}$$

where $L_{\mathrm{recon}}=\frac{1}{N}{\sum_{i=1}^{N}\left\|x_{i}-{\hat{x}}_{i}\right\|}_{2}^{2}$ denotes the reconstruction loss, *L_SVDD_* denotes the SVDD hypersphere loss, and *λ*_1_, *λ*_2_, and *α* denote the weight coefficients of the reconstruction loss, the SVDD loss, and the alignment loss, respectively. In each iteration, the gradients of all the loss terms are computed uniformly and used to update all the trainable parameters of the model through backpropagation, thereby ensuring that the model adheres to the physical priors while fitting the limited training samples and constructs a compact decision boundary that is data adaptive, physically plausible, and robust against noise in regions with sparse data.

In addition to the loss function design, the anomaly threshold serves as another key component for the DASVDD model in performing anomaly detection tasks. The anomaly threshold is a key criterion for distinguishing normal samples from abnormal samples; essentially, it is a critical value set by humans or algorithms, used to convert the continuous anomaly scores output by the model into discrete classification results. The anomaly threshold of the proposed DASVDD model is determined based on the anomaly scores of the validation set. First, the probability distribution of the scores is estimated and fitted through kernel density estimation, as follows:

(31) $$f\left(s\right)=\frac{1}{\mathrm{Mh}}\sum_{j=1}^{M}K\left(\frac{s-s_{j}}{h}\right)$$

where *K*(⋅) is the Gaussian kernel function satisfying $\int_{-\infty }^{+\infty }K\left(x\right)\mathrm{dx}=1$, *s_j_* denotes the anomaly score samples in the validation set, *M* is the number of samples, and *h* is the kernel bandwidth, which is automatically determined by Scott’s rule according to the sample size so as to satisfy the asymptotic optimality condition of kernel density estimation. Then, the threshold is solved based on the cumulative distribution function. The target confidence level is set to *δ* = 99.7%, and the threshold *ϕ* needs to satisfy the following integration condition of the cumulative distribution function (CDF):

(32) $$\int_{-\infty }^{\phi }f\left(s\right)\mathrm{ds}=\delta$$

In practice, the threshold *ϕ* is obtained by numerically solving Equation (32) via the bisection method within a bounded interval determined by the extremes and standard deviation of the validation scores. The confidence level is set to *δ* = 99.7%, which corresponds to the conventional three-sigma (3*σ*) criterion in statistical process control. This level provides a balanced trade-off: a lower threshold absorbs excessive normal fluctuations and inflates false alarms, whereas a higher threshold misses incipient anomalies whose scores have not yet fully separated from the normal distribution. For each test sample, if its anomaly score exceeds *ϕ*, it is classified as anomalous; otherwise, it is deemed normal. This criterion avoids manual specification of the threshold value itself. Although the confidence level is chosen based on the conventional 3*σ* criterion, it remains a user-defined parameter; its influence on detection performance is therefore examined in the sensitivity analysis below.

### 2.3. Physically-Informed Bidirectional Adjustment Reconstruction Error

**Figure 5:**
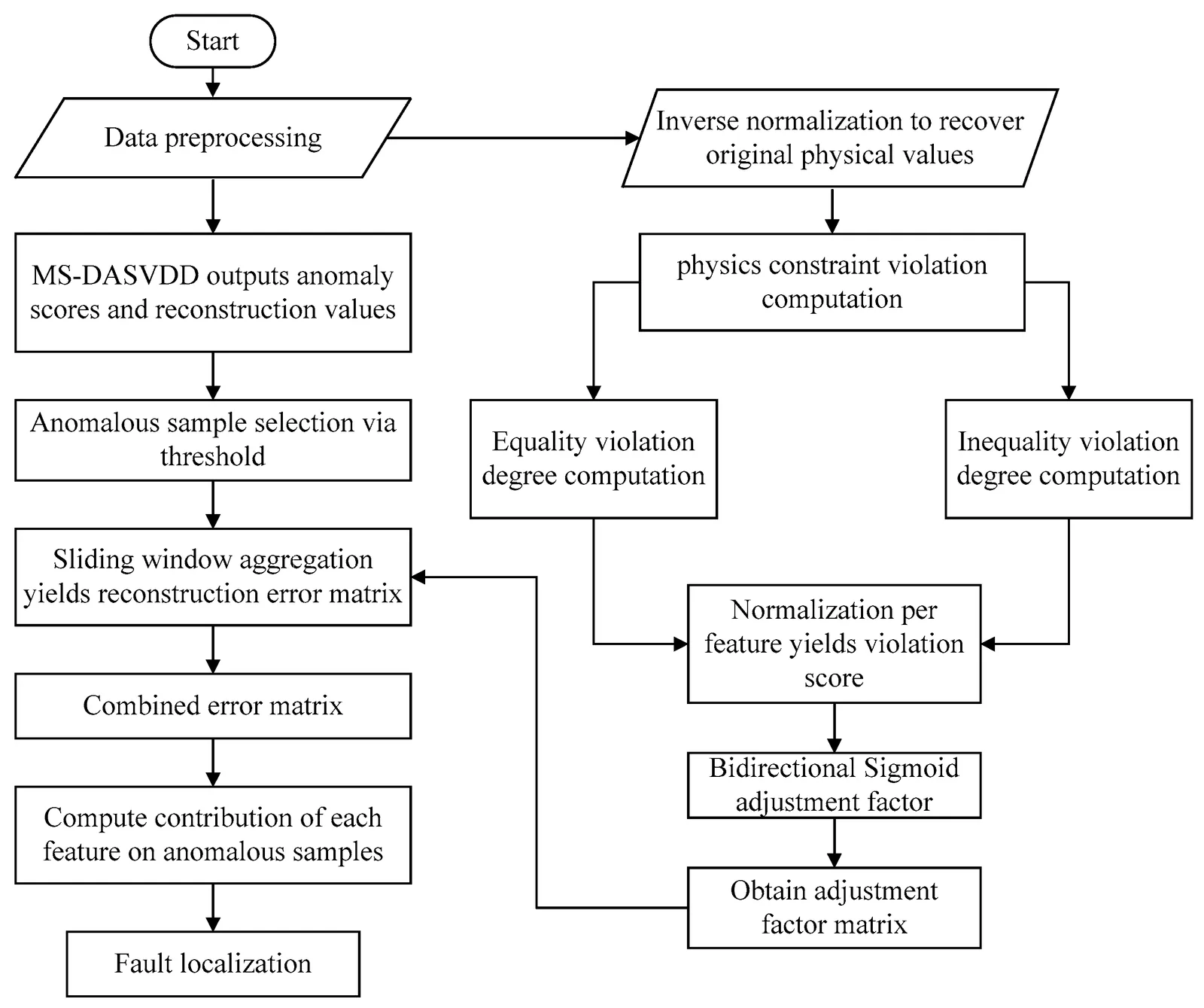
Flowchart of the operational process of fault localization.

Using the autoencoder embedded in the trained MS-DASVDD model as the reconstruction backbone, the reconstruction values of the test samples are obtained via forward propagation, and the relative reconstruction error of each sample at each feature dimension is derived through sliding window aggregation. To further improve the identifiability of fault features, a physically informed bidirectional adjustment reconstruction error is developed. The degree of violation of the domain physical rules by each feature is first quantified on the original input after inverse normalization, i.e., on the true physical quantities, and the reconstruction errors are then differentially adjusted according to this violation degree. Specifically, a suppressive reward factor is applied to the constrained features that comply with the physical rules so as to attenuate their responses, an amplifying penalty factor is imposed on the constrained features that violate the physical rules so as to amplify their responses, and the features not covered by the physical constraints are left unchanged. The flowchart of the fault localization process is shown in Figure 5.

Step 1: Data preparation. Let the test sample set be denoted by $X=\left\{x_{1}\right.,x_{2}\ldots ,\left.x_{N}\right\},x_{i}\in R^{D_{f}}$, where *N* is the number of samples and *D_f_* is the feature dimensionality. The feature index set covered by the physical constraints is defined as *Ι*_C_ ⊆ {1, 2, …, *D*}, which includes the pitch angles (pitch 1, pitch 2, and pitch 3), the pitch motor temperatures (motor temp 1, motor temp 2, and motor temp 3), the nacelle temperatures (cabinet, inner, and outer), the rotor speed, the generator speed, the gearbox high-speed and low-speed bearing temperatures, and the bearing temperature at the non-drive end of the generator; the remaining features are unconstrained, and their adjustment factors are always fixed at 1.

Step 2: Window aggregated reconstruction error computation. For each sample *x_i_*, the relative reconstruction error with respect to the reconstruction output ${\hat{x}}_{i}$ of the autoencoder is computed by sliding window aggregation. Let *L* denote the window length and *S* the step size, and let *W_i_* denote the set of windows covering sample *i*. Within each window *w* ∈ *W_i_*, the relative reconstruction error of sample *i* at feature *j* is defined as:

(33) $$e_{i,j}^{\left(w\right)}=\left|{\bar{x}}_{i,j}-{\hat{\bar{x}}}_{i,j}\right|,j=1,\ldots ,D$$

where |·| denotes the absolute value operation, ${\bar{x}}_{i,j}$ denotes the input feature after MinMax normalization, and ${\hat{\bar{x}}}_{i,j}$ is the reconstruction value produced by the autoencoder. After window aggregation, the average relative reconstruction error of sample *i* at feature *j* is given by:

(34) $${\bar{e}}_{i,j}=\frac{1}{K_{i}}\sum_{w\in W_{i}}{e_{i,j}}^{\left(w\right)}$$

where *K_i_* = |*W_i_*| is the number of windows covering sample *i*.

Step 3: Quantification of physical constraint violations (based on the original input). Unlike the common practice of computing physical violations on the reconstruction output, a key design of this work is that the degree of physical constraint violation is evaluated on the original input after inverse normalization, i.e., on the true physical quantities, rather than on the reconstruction output. This is because anomalies physically originate from the deviation of the original input from the physical laws of the turbine, whereas the reconstruction output has already been corrected by the physics regularization and may conceal the true violation information. Let $\varphi_{i,j}^{\left(k\right)}\geq 0$ denote the degree to which sample *i* violates the *k*-th physical constraint at feature *j*. The six physical constraints, namely pitch angle consistency, motor temperature consistency, nacelle temperature ordering, rotor speed ratio, pump state, and fan state, are uniformly expressed as:

(35) $$\Phi_{i,j}=\sum_{k=1}^{K}\omega_{k}\cdot \varphi_{i,j}^{\left(k\right)}$$

where *K* is the total number of physical constraints (*K* = 6), $\varphi_{i,j}^{\left(k\right)}$ is the violation value of the *k*-th constraint at feature *j*, and *ω_k_* is the weight of the *k*-th constraint.

Step 4: Normalized violation score. For each constrained feature *j* ∈ *Ι*_C_, the violation value is normalized along the feature dimension to [0, 1], yielding the normalized violation score:

(36) $$\nu_{i,j}=\frac{\Phi_{i,j}}{\underset{i}{\max}\Phi_{i,j}+\delta }$$

where *δ* is a small positive constant used to prevent division by zero. For unconstrained features *j* ∉ *Ι*_C_, *ν_i_*_,*j*_ is set to 0.

Step 5: Design of the bidirectional adjustment factor. The adjustment factor *a_i_*_,*j*_ ∈ [*a*_min_, *a*_max_] is defined as:

(37) $$a_{i,j}=\left\{\begin{array}{l} \min\left(a_{\max},\max\left(a_{\min},1+\rho \cdot \sigma \left(\psi \left(v_{i,j}-ο\right)\right)-\xi \cdot \left(1-\sigma \left(\psi \left(v_{i,j}-ο\right)\right)\right)\right)\right),j\in {Ι}_{C}\\ 1,j\notin {Ι}_{C} \end{array}\right.$$

where $\sigma \left(z\right)=\frac{1}{1+e^{-z}}$ is the Sigmoid function, *ο* is the adjustment threshold that controls the triggering point of penalty and reward, *ψ* is the steepness parameter that controls the smoothness of the adjustment curve, *ρ* is the penalty intensity, *ξ* is the reward intensity, and *a*_min_ and *a*_max_ are the lower and upper bounds of the adjustment factor, enforced through the clipping operation embedded in Equation (37). When *v_i_*_,*j*_≫*ο*, we have *a_i_*_,*j*_ → 1 + *ρ > *1, which penalizes the features that violate the physical laws by amplifying their reconstruction errors; when *v_i_*_,*j*_≪*ο*, we have *a_i_*_,*j*_ → 1 − *ξ < *1, which rewards the features that comply with the physical laws by shrinking their reconstruction errors. The neutral point at which *a_i_*_,*j*_ = 1 occurs at $v_{i,j}=ο+\frac{1}{\psi }\ln\left(\xi /\rho \right)$. When *ξ* = *ρ*, this reduces to *v_i_*_,*j*_ = *ο*, i.e., the threshold coincides with the neutral point. When *ξ* ≠ *ρ*, the neutral point shifts away from *ο* by $\frac{1}{\psi }\ln\left(\xi /\rho \right)$. In particular, when *ξ > ρ* (reward dominates), the neutral point lies above the threshold, so that a larger fraction of samples fall into the reward region. This is a deliberate design choice for datasets with few physical violations: it applies strong regularization to compliant features while reserving penalty for the most extreme violations.

Step 6: Combined error and fault localization. The combined error used for fault localization is defined as:

(38) $$s_{i,j}={\bar{e}}_{i,j}\cdot a_{i,j}$$

The global fault contribution of feature *j* is given by:

(39) $$S_{j}=\frac{1}{N_{a}}\sum_{i\in A}s_{i,j}$$

where *A* is the set of anomalous samples used for offline evaluation, i.e., the samples whose anomaly scores exceed the 99.7% statistical threshold of the DASVDD scores and occur after the onset of the true anomaly. The true anomaly onset is employed only to exclude historical false alarms, ensuring a fair comparison of fault localization performance across different methods. Online deployment, where the true onset is unavailable, is beyond the scope of this work and requires a data-driven triggering mechanism (e.g., consecutive threshold-exceeding), which is left for future research. *N_a_* = |*A*| is the number of anomalous samples. The fault features are ranked according to *S_j_* in descending order, and a larger *S_j_* indicates that the corresponding feature is more likely to be the root cause of the fault. Through the above bidirectional adjustment, constrained features that violate physical rules are promoted in ranking due to penalized amplification of their errors, whereas those that are physically consistent are demoted due to rewarded suppression of their errors, while unconstrained features remain unchanged. Consequently, the contrast and interpretability of fault localization are effectively enhanced.

## 3. Results

### 3.1. Effectiveness Validation of Explicit Algebraic Physics Constraints

#### 3.1.1. Case One: Wind Turbines in the 30 MW Wind Farm

The wind turbine suffered a fault on 26 December 2025. Subsequent inspections suggested that abnormal large particle contaminants, including metallic wear debris, sealing material fragments, and sludge agglomerates, may have accumulated inside the gearbox, or that the lubricating oil had undergone degradation. These contaminants gathered in the oil circulation path and partially obstructed the oil filter, causing a drastic increase in filter flow resistance and a consequent rapid rise in inlet pressure. The filter obstruction led to insufficient lubricating oil circulation. The reduced oil flow through the cooler impaired the dissipation of heat generated by the gearbox, significantly lowering the cooling efficiency and ultimately resulting in a persistently elevated gearbox oil temperature under load.

For Case one, to verify the efficacy of embedding turbine intrinsic physical relationships into the network, an explicit algebraic physics constraint validation framework based on an autoencoder was established, in which all 24 operational variables served as input features. All datasets were sampled at a 1 min interval. For the source-domain dataset, samples violating physical laws were removed before pretraining. For the target-domain dataset, only a portion of clearly invalid samples (e.g., samples with zero wind speed and zero power output) were removed, while the remaining samples were retained to preserve the real operating distribution of the target turbine. For the reconstruction-fidelity validation dataset, samples violating physical laws were discarded before evaluation. The source-domain dataset was then split with 80% of its samples used for pretraining, while the target-domain dataset was divided into a training set and an independent validation set, with 85% of the samples used to train the normal behavior model. The dataset configurations for source-domain pretraining, target-domain fine-tuning, and reconstruction-fidelity validation are summarized in Table 4.

Among the six physical constraints, the generator cooling fan constraint is the only one that involves temporal dependency. It requires that, after a delay, the non-drive-end bearing temperature decreases while the generator cooling fan is continuously ON and increases while it is continuously OFF. Since this constraint is evaluated on temperature differences between consecutive time steps, the model input must be organized as sliding windows rather than independently shuffled samples. Let *L* and *S* denote the window length and step size, respectively; the *k*-th window covers [*kS*, *kS* + *L* − 1]. During training, only the window order is shuffled, while the intra-window temporal order is preserved, so that the generator cooling fan constraint remains well-defined. Shuffling at the sample level would break temporal continuity and render the temperature differences physically meaningless. For consistency, the same window length *L* and step size *S* are used across the training, validation, testing, and fault-localization stages. In particular, the fault-localization stage computes window-averaged reconstruction errors using the same window partitioning, ensuring that reconstruction errors and physics-constraint violations are statistically comparable across all stages. For Case one, the parameter settings of the autoencoder and the six physical constraints are summarized in Table 5 and Table 6, respectively.

Based on this, the reconstruction-fidelity validation dataset was employed to evaluate both the effectiveness of physical constraints and the reconstruction fitting performance. Furthermore, nine experimental configurations were designated as A through I: A was the baseline without physics constraints; B through G corresponded to the models with only the pitch angle, motor temperature, nacelle temperature ordering, rotor speed ratio, gearbox cooling water pump, and generator cooling fan constraints, respectively; H was the model with all six constraints combined; and I was the model combining all six constraints with transfer learning. The results validating the effectiveness of embedding explicit algebraic physical relationships were presented in Table 7, where the root mean square error (RMSE), mean absolute error (MAE), and weighted absolute percentage error (WAPE) were used as evaluation metrics to compare configurations A through I.

(40) $$\mathrm{RMSE}=\sqrt{\frac{1}{n}\sum_{i=1}^{n}{\left(y_{i}-{\hat{y}}_{i}\right)}^{2}}$$

(41) $$\mathrm{MAE}=\frac{1}{n}\sum_{i=1}^{n}\left|y_{i}-{\hat{y}}_{i}\right|$$

(42) $$\mathrm{WAPE}=\frac{\sum_{i=1}^{m}\left|y_{i}-{\hat{y}}_{i}\right|}{\sum_{i=1}^{m}y_{i}}\times 100\%$$

where *y_i_* denotes the ground truth, and ${\hat{y}}_{i}$ denotes the corresponding reconstruction.

**Figure 6:**
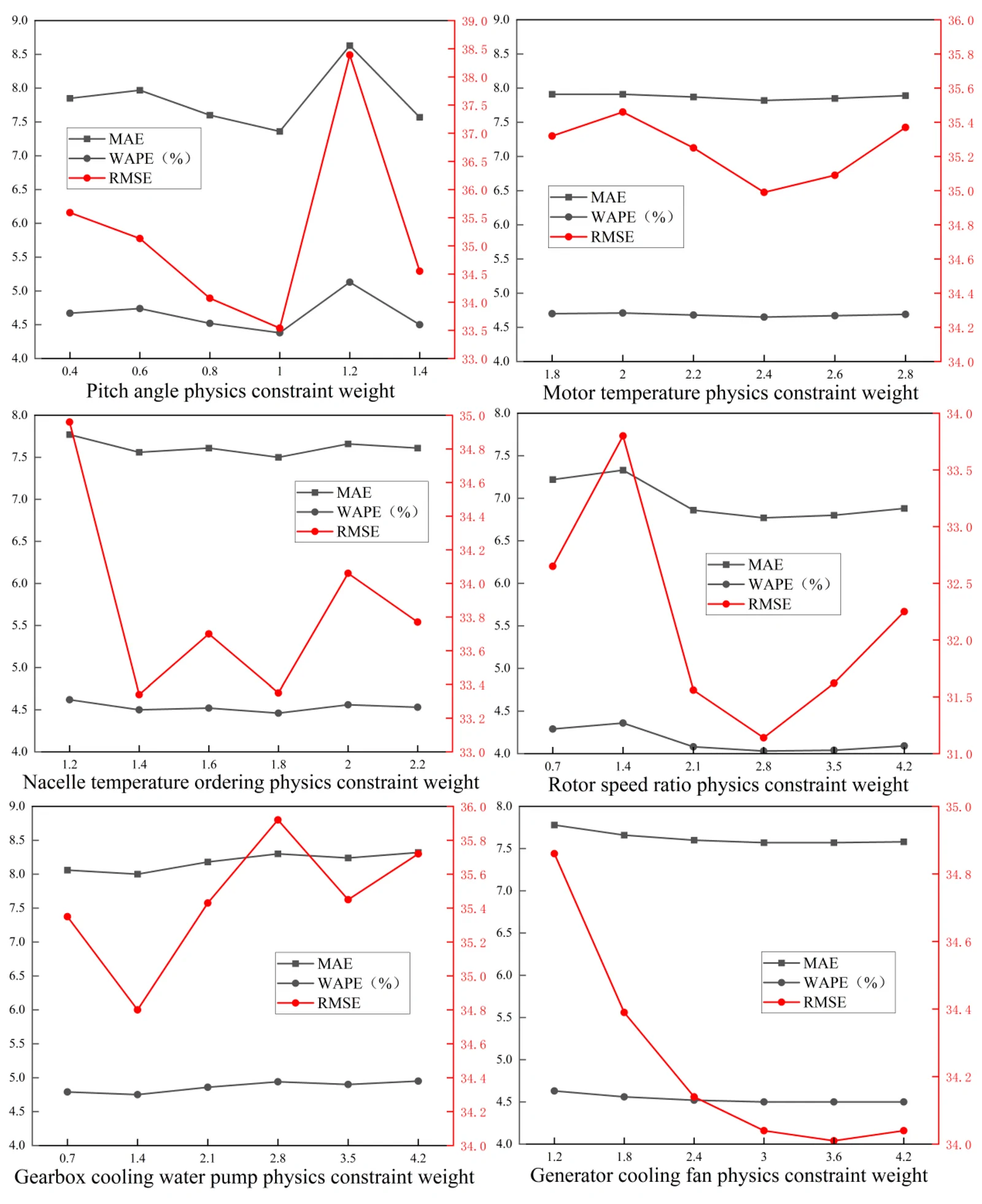
Sensitivity analysis of individual physical constraint weights for Case one.

To quantitatively investigate how physical constraint loss weight affects model diagnostic performance, we performed single-factor sensitivity analysis for each of the six physical constraints. For every individual constraint, all other physical regularizers were deactivated, while the target constraint loss weight was swept over a reasonable hyperparameter range. Reconstruction performance was quantified using MAE, RMSE, and WAPE. As shown in the sensitivity analysis curves (Figure 6), model performance exhibits clear non-monotonic trends with varying constraint weights. Too small a weight fails to introduce adequate physical priors and yields marginal performance gains. Conversely, an overly large weight dominates the total loss, excessively suppresses the data-driven reconstruction term, and introduces physical bias, degrading diagnostic accuracy. For each constraint, the optimal weight is chosen at the inflection point where MAE, RMSE and WAPE reach their minima simultaneously. This setting achieves a favorable trade-off between data-driven fitting and physical prior regularization.

As illustrated by the evaluation metrics of various models in Table 7, the physical constraints are effective in the final model, thereby validating the effectiveness of embedding explicit algebraic physical relationships. However, activating a single physical constraint with the optimal weight determined by sensitivity analysis only yields limited reductions in RMSE, MAE and WAPE. This is because individual physical regularization only imposes constraints on its corresponding physical dimension, while anomalous samples that violate physical laws in the remaining five dimensions remain unregulated. Each of the six physical constraints corresponds to an independent optimization objective with intricate coupling interactions, which deliver both synergistic enhancement and mutual trade-off effects. Accordingly, the joint application of all six physical constraints cannot achieve a substantial improvement in reconstruction metrics compared with single-constraint scenarios, but this coupled configuration enables the best overall performance of the model. It can effectively regularize physical features across all dimensions and achieve a reasonable balance between data-driven fitting and multi-physical prior regularization. Ultimately, the introduction of transfer learning further optimizes the model’s generalization performance and achieves the best reconstruction results.

#### 3.1.2. Case Two: Wind Turbines in the 40 MW Wind Farm

The wind turbine experienced a fault on 18 December 2025. Subsequent investigations revealed that an abnormal alarm indicating an excessive nacelle ambient temperature was triggered during turbine operation. On-site verification and sensor status diagnostics confirmed that this alarm was not caused by an actual temperature rise within the nacelle, but rather by a malfunction of the nacelle ambient temperature sensor. Specifically, performance drift or structural damage to the sensor led to distorted temperature readings, outputting erroneously high values that significantly deviated from the actual operating conditions, and ultimately activating the turbine’s over-temperature protection alarm.

For Case two, to verify the efficacy of embedding turbine intrinsic physical relationships into the network, an explicit algebraic physics constraint validation framework based on an autoencoder was established, in which all 24 operational variables served as input features. All datasets were sampled at a 1 min interval. For the source-domain dataset, samples violating physical laws were removed before pretraining. For the target-domain dataset, only a portion of clearly invalid samples (e.g., samples with zero wind speed and zero power output) were removed, while the remaining samples were retained to preserve the real operating distribution of the target turbine. For the reconstruction-fidelity validation dataset, samples violating physical laws were discarded before evaluation. The source-domain dataset was entirely used for pretraining, while the target-domain dataset was divided into a training set and an independent validation set, with 85% of the samples used to train the normal behavior model. The dataset configurations for source-domain pretraining, target-domain fine-tuning, and reconstruction-fidelity validation are summarized in Table 8.

This reconstruction-fidelity validation dataset was used to assess constraint effectiveness and reconstruction fidelity. Nine configurations (A–I) were designed: A was the baseline without constraints; B–G each incorporated a single constraint (pitch angle, motor temperature, nacelle temperature ordering, rotor speed ratio, gearbox cooling water pump, and generator cooling fan, respectively); H combined all six constraints; and I further integrated transfer learning. The parameter settings of the autoencoder and the six physical constraints are summarized in Table 9 and Table 10, respectively. Based on these settings, the evaluation results of the nine configurations are summarized in Table 11, with RMSE, MAE, and WAPE as evaluation metrics.

**Figure 7:**
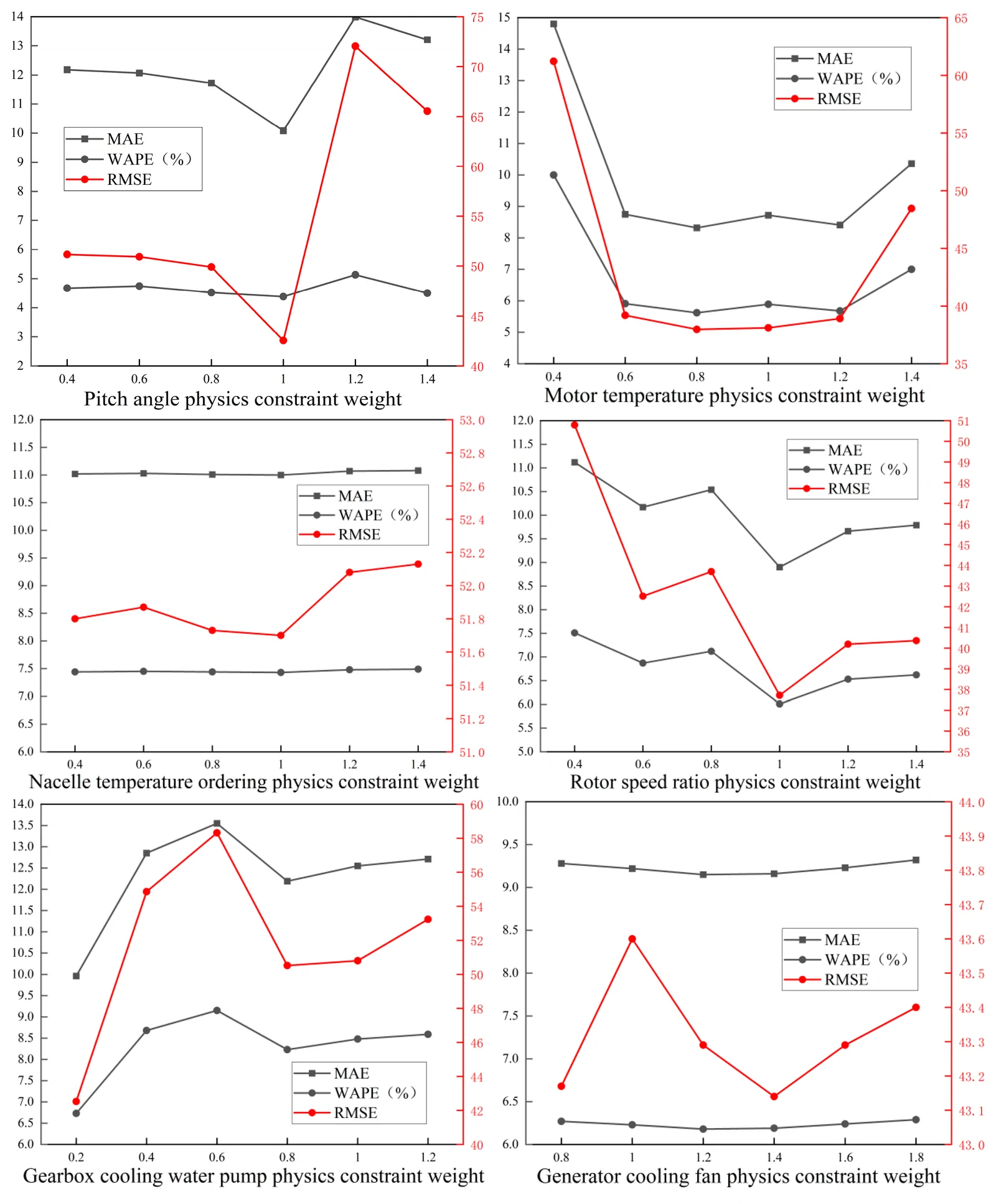
Sensitivity analysis of individual physical constraint weights for Case two.

To clarify how the physical constraint loss weight affects diagnostic performance, a single-factor sensitivity study was performed on each of the six constraints. For each one, the other regularizers were disabled, and only its weight was varied over a suitable range, with MAE, RMSE, and WAPE used to evaluate reconstruction quality. The sensitivity curves (Figure 7) show a clear non-monotonic trend: a too-small weight gives insufficient physical priors and marginal gains, while a too-large weight dominates the total loss, over-constrains the data-driven term, and introduces physical bias that degrades diagnostic accuracy. The optimal weight is thus selected at the point where MAE, RMSE, and WAPE simultaneously reach their minima, yielding a favorable trade-off between data-driven fitting and physical prior regularization.

The metrics in Table 11 confirm that the physical constraints are effective in the final model, validating the embedding of explicit algebraic physical relationships. However, a single constraint with its sensitivity-optimized weight only modestly reduces RMSE, MAE, and WAPE, as it regulates only its own physical dimension while anomalies in the other five remain unconstrained. Although the six constraints are coupled with both synergistic and trade-off effects, their joint use does not substantially improve reconstruction metrics over single-constraint cases, but it does achieve the best overall performance by balancing data-driven fitting and multi-physical prior regularization, with transfer learning further improving generalization and reconstruction.

### 3.2. Anomaly Detection Results

#### 3.2.1. Training and Inference Procedures of MS-DASVDD

The complete training and inference procedures of the proposed MS-DASVDD framework are summarized in Algorithm 1 and Algorithm 2, respectively. The two case studies share the same algorithmic procedure and differ only in the dataset and hyperparameter settings.
**Algorithm 1**: Training stage of MS-DASVDDInput:        Source-domain dataset (with pump/fan status columns)        Target-domain dataset, split into *Dtrain* (85% training) and *Dval* (15% validation)        Physical constraint set *c* = {*c*_1_, …, *c*_6_}        Window length *L* = 60, step size *S* = 30        Autoencoder layer sizes: [24, 16, 8, 4, 8, 16, 24]        Hyperparameters: *λ*_1_, *λ*_2_, *α*, *ν*, *γ*, batch size *B*, Epoch size 1, Epoch size 2,                                        learning rate, frozen encoder layers, early-stopping patience *P*Output:        Trained model *M*1: Exclude pump/fan status columns; normalize features with Min–Max scaler2: Construct sliding windows (*L*, *S*); shuffle window order only3: Build the autoencoder, SVDD layer, and loss terms4: Stage 1: Source-domain pre-training5: for *epoch* = 1 to Epoch size 1 do6:        for each window batch *B* in Source-domain dataset do7:                Compute $L_{\mathrm{total}}=\lambda_{1}L_{\mathrm{recon}}+L_{\mathrm{phys}}^{\mathrm{direct}}+\alpha L_{\mathrm{align}}+\lambda_{2}L_{\mathrm{svdd}}$
8:                Update all trainable parameters by Adam with learning rate9:        end for10: end for11: Save pre-trained weights *θ_s_*12: Stage 2: Target-domain fine-tuning13: Load *θ_s_*; freeze the first *k* encoder layers14: for *epoch* = 1 to Epoch size 2 do15:        for each window batch *B* in *Dtrain* do16:                Compute *L_total_* as in line 717:                Update trainable parameters by Adam18:        end for19:        if validation loss does not improve for *P* epochs then20:                Early stop; restore the best weights21:        end if22: end for23: Unfreeze all encoder layers24: return *M*

**Algorithm 2**: Inference stage of MS-DASVDDInput:        Trained model *M*        Threshold-calibration dataset *Dval* (15% of target domain)        Test dataset *Dtest*        Window length *L* = 60, step size *S* = 30        Confidence level *δ*        Aggregation strategy: first-covering-window        (for overlapping samples, keep the score from the first window that covers them)Output:        Per-sample anomaly scores for *s*_*test*        Detected anomaly indices        Ranked fault features1: Exclude pump/fan status columns; normalize using the training scaler2: Construct sliding windows (*L*, *S*) 3: Threshold calibration4: for each window *w* in *Dval* do5: Compute the anomaly score *s*(*w*) of window *w* using *M*6: end for7: Aggregate window scores into per-sample scores *x_i_*, *i* = 1, …, *n*    (aggregation = ‘first’: each sample keeps the score from the first    covering window; uncovered samples are filled by nearest neighbor)8: Fit kernel density estimate (KDE) *f*(*x*) on {*x_i_*}9: Solve *ϕ* such that *CDF*(*ϕ*) = *δ*10: Test-set inference11: for each window *w* in *Dtest* do12: Compute the anomaly score *s*(*w*) of window *w* using *M*13: end for14: Aggregate window scores into per-sample scores *s*_*test*      using the same ‘first’ strategy15: *A* ← {*i*|*s*_*test*[*i*] *> ϕ*}16: Fault localization17: for each anomaly sample *i* in *A* do18:         ${\bar{e}}_{i,j}$ ← window-averaged reconstruction error of sample *i*19:         *v_i_*_,*j*_ ← physics-violation score computed on the original (non-reconstructed) input of sample *i*20:         *a_i_*_,*j*_ ← bidirectional adjustment factor (penalty/reward)21:         $s_{i,j}={\bar{e}}_{i,j}\times a_{i,j}$
22: end for23: For each feature *j*, compute the global fault contribution *S_j_* by averaging *s_i_*_,*j*_ over all *i* ∈ *A*24: Rank features by *S_j_* in descending order25: return *s*_*test*, *A*, ranked fault features

#### 3.2.2. Case One: Wind Turbines in the 30 MW Wind Farm

For the MS-DASVDD anomaly detection model, both the hyperparameter configurations of its autoencoder module and the parameter settings of the six physical constraints are kept identical to those adopted in the explicit algebraic physics constraint expressibility validation mechanism. Regarding dataset partitioning, the source-domain dataset for transfer learning does not follow the same partitioning strategy, whereas the target-domain training dataset adopts a partitioning strategy consistent with that used in the validation mechanism. These settings ensure that the introduced physical constraints can function effectively within the MS-DASVDD framework. Furthermore, the remaining 15% of the target-domain dataset is leveraged to determine the anomaly detection threshold, while valid SCADA samples serve as the test set for model evaluation. The detailed configurations of all datasets are summarized in Table 12, including their collection dates, sample sizes, health states, turbine identity, faulty samples and purpose. The main parameters of the MS-DASVDD model are listed in Table 13.

To further evaluate the sensitivity of the anomaly detection performance to the choice of the confidence level, a series of thresholds derived from different confidence levels are examined, and the corresponding anomaly detection results are summarized in Table 14. This analysis aims to verify whether the selected 99.7% confidence level lies within a favorable operating region where both false alarms and missed detections are well controlled.

As shown in Table 14, the detection performance exhibits a clear precision–recall trade-off as the confidence level varies. At low confidence levels (95.0% and 99.0%), the threshold is sufficiently low to capture all anomalous samples (Recall = 1.0000), yet the resulting false alarm rate is excessively high, with Precision dropping to 0.2709 and 0.5047, respectively, and the F1 score falling to 0.4263 and 0.6708. As the confidence level increases to 99.5%, Precision improves to 0.6731 while Recall remains at 0.9813, yielding an F1 score of 0.7985. At *δ* = 99.7%, the threshold strikes the optimal balance, achieving a Precision of 0.8696 and a Recall of 0.9346, with the F1 score peaking at 0.9009. Further raising the confidence level to 99.9% increases Precision to 0.9880, but at the cost of a pronounced decline in Recall to 0.7664, as the threshold begins to exclude incipient anomaly samples whose scores have not yet reached the higher cutoff. The F1 score consequently drops to 0.8632. This trend confirms that the 99.7% threshold, derived a priori from the 3σ statistical criterion on the validation set, lies within the favorable operating region where both false alarms and missed detections are well controlled, and that the selected confidence level is well justified.

**Figure 8:**
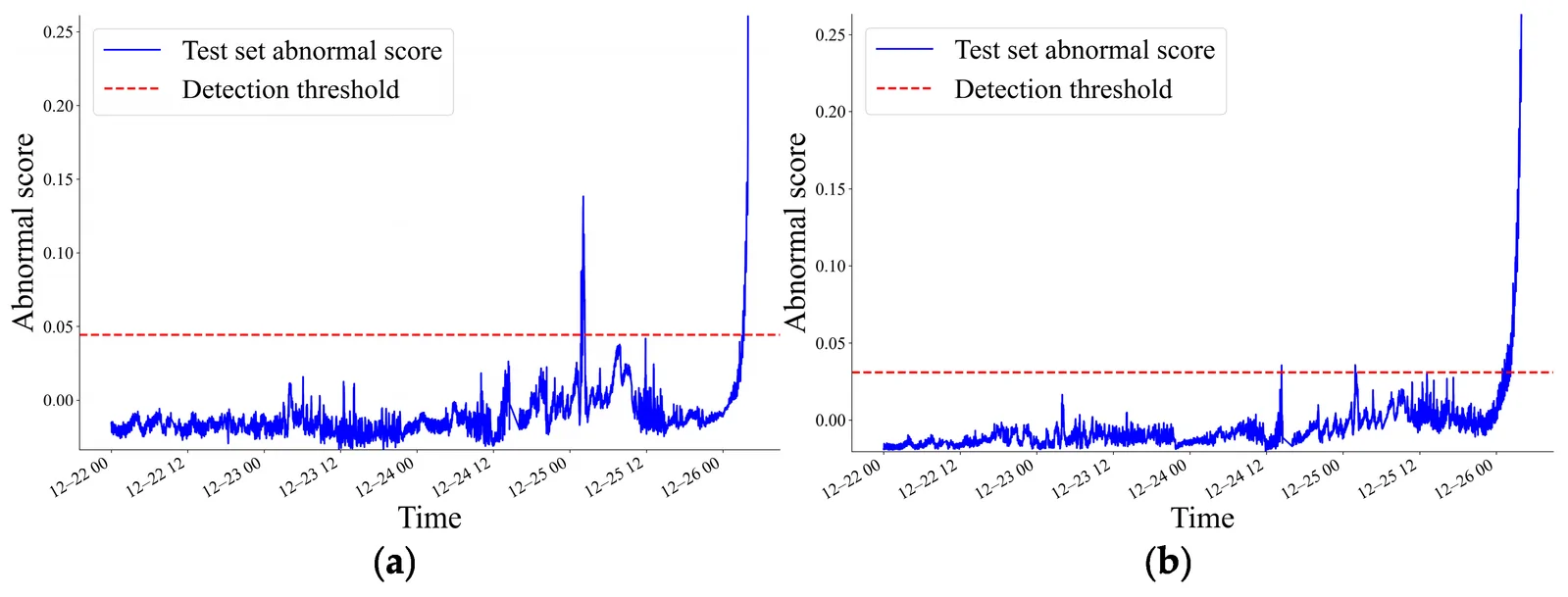
Anomaly detection results: (**a**) Anomaly score for DASVDD anomaly detection; (**b**) Anomaly score for MS-DASVDD anomaly detection.

Based on the sensitivity analysis above, the 99.7% confidence level is adopted as the operating threshold for the subsequent anomaly detection evaluation. Under this threshold, the anomaly detection performance of different models is further compared, and the results are summarized in Table 15 using Precision, Recall, F1 score, and Accuracy [28]. The corresponding anomaly detection outcomes derived from the anomaly scores are visualized in Figure 8.

Table 15 demonstrates that MS-DASVDD outperforms the baseline DASVDD and its variants, achieving the highest precision, recall, and F1 score. The temporal comparison results in Figure 8 further indicate that, upon incorporating physical constraints, latent space alignment constraints, and the transfer learning strategy, MS-DASVDD effectively suppresses noise interference that violates physical laws in the training set, reduces the model’s dependence on training sample size, and achieves better separation between the anomaly scores of normal and faulty samples on the test set, thereby mitigating both false negatives and false positives and enhancing anomaly detection performance. Specifically, several spurious spikes in the anomaly scores shown in Figure 8a are noticeably attenuated after suppression, as illustrated in Figure 8b.

#### 3.2.3. Case Two: Wind Turbines in the 40 MW Wind Farm

For the MS-DASVDD anomaly detection model, both the hyperparameter configurations of its autoencoder module and the parameter settings of the six physical constraints are kept identical to those adopted in the explicit algebraic physics constraint expressibility validation mechanism. Regarding dataset partitioning, the source-domain dataset for transfer learning does not follow the same partitioning strategy, whereas the target-domain training dataset adopts a partitioning strategy consistent with that used in the validation mechanism. These settings ensure that the introduced physical constraints can function effectively within the MS-DASVDD framework. Furthermore, the remaining 15% of the target-domain dataset is leveraged to determine the anomaly detection threshold, while valid SCADA samples serve as the test set for model evaluation. The detailed configurations of all datasets are summarized in Table 16, including their collection dates, sample sizes, health states, and purpose. The main parameters of the MS-DASVDD model are listed in Table 17.

To further evaluate the sensitivity of the anomaly detection performance to the choice of the confidence level, a series of thresholds derived from different confidence levels are examined, and the corresponding anomaly detection results are summarized in Table 18. This analysis aims to verify whether the selected 99.7% confidence level lies within a favorable operating region where both false alarms and missed detections are well controlled.

As shown in Table 18, for this case, the F1 score peaks at 99.5% (0.9266) and remains at a comparable level at 99.0% (0.9258). At the selected confidence level of *δ* = 99.7%, the F1 score is 0.8600, with Precision reaching 1.0000 and Recall at 0.7544. The slightly lower F1 score at 99.7% is attributed to the higher threshold excluding a portion of incipient anomaly samples; however, the resulting perfect precision (zero false alarms) is particularly desirable in practical wind turbine condition monitoring, where unnecessary false alarms can trigger costly on-site inspections and turbine downtime. It should be emphasized that the 99.7% threshold was determined exclusively from the validation set based on the 3σ statistical criterion, without accessing any test set labels. The sensitivity results across 99.0–99.9% demonstrate that the proposed method maintains F1 scores above 0.80, indicating acceptable robustness to the confidence level choice.

**Figure 9:**
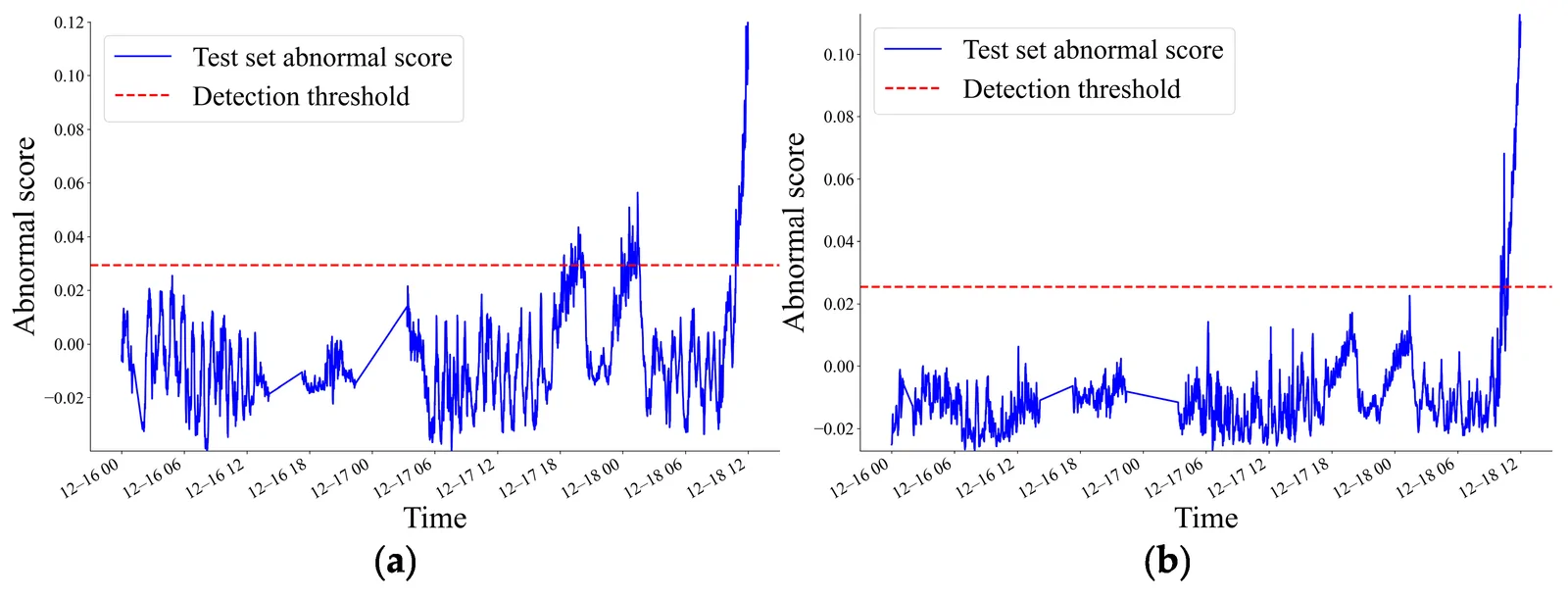
Anomaly detection results: (**a**) Anomaly score for DASVDD anomaly detection; (**b**) Anomaly score for MS-DASVDD anomaly detection.

Based on the sensitivity analysis above, the 99.7% confidence level is adopted as the operating threshold for the subsequent anomaly detection evaluation, owing to its perfect precision and acceptable F1 score. Under this threshold, the anomaly detection performance of different models is further compared, and the results are summarized in Table 19 using Precision, Recall, F1 score, and Accuracy. The corresponding anomaly detection outcomes obtained from the anomaly scores are illustrated in Figure 9.

As evidenced by Table 19, MS-DASVDD surpasses the baseline DASVDD and its variants, yielding the highest precision, recall, and F1 score. The temporal comparison shown in Figure 9 further reveals that, by jointly integrating physical constraints, latent space alignment constraints, and transfer learning, MS-DASVDD is able to substantially suppress the noise interference that violates physical laws in the training set, diminish its dependence on training sample size, and achieve stronger separability of anomaly scores between normal and faulty samples on the test set. This in turn reduces both false negatives and false positives, thereby improving the overall anomaly detection performance. In particular, several spurious spikes in the anomaly scores presented in Figure 9a are markedly attenuated after suppression, as shown in Figure 9b.

### 3.3. Fault Localization Results

The fault localization parameters for Case one and Case two are summarized in Table 20. Note that the reward weight *ξ*, the penalty weight *ρ*, and the violation threshold *ο* differ between the two cases. This is because the two cases exhibit different anomaly characteristics: in Case one, the anomalous features are predominantly those that comply with the physical constraints but deviate from the normal operating distribution, so a larger reward weight is adopted to suppress the interference from non-fault features; in Case two, the anomalies are more likely to manifest as violations of the physical constraints, so a relatively larger penalty weight is adopted to amplify the fault-related features. The effectiveness of these parameter settings is further verified by the fault localization results presented below.

**Figure 10:**
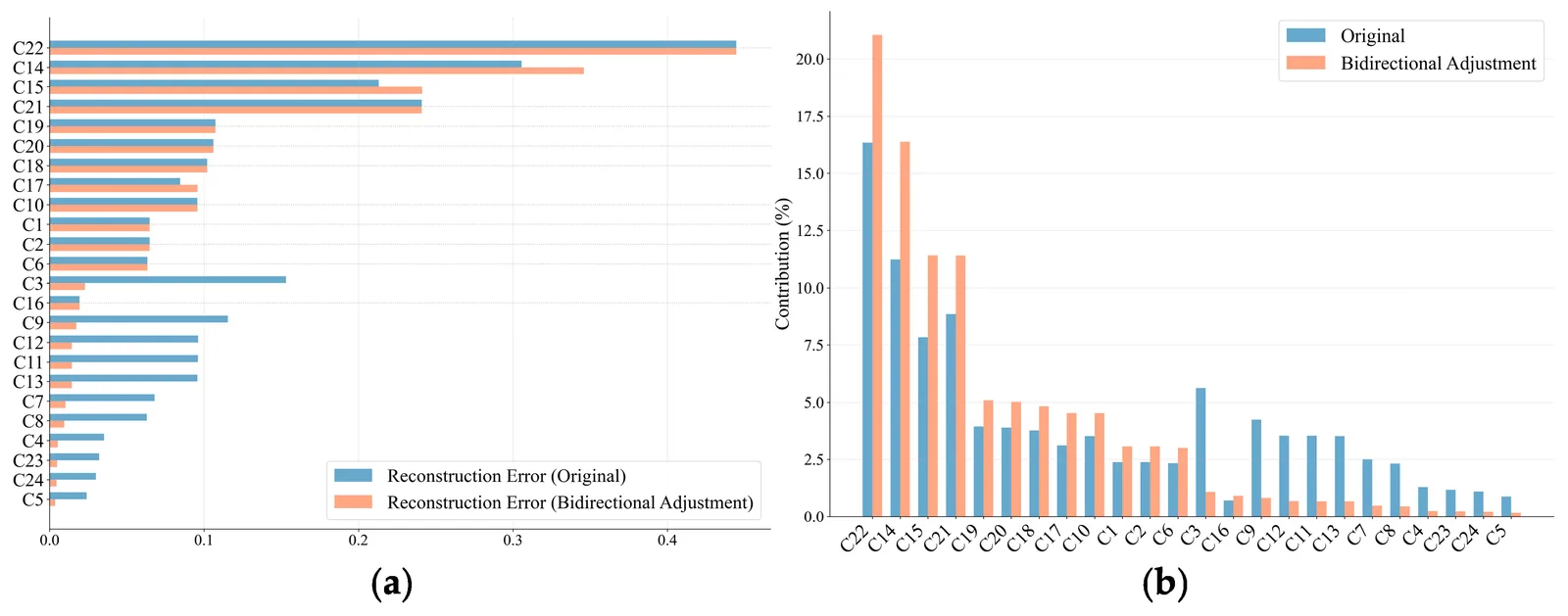
Fault localization results for Case one: (**a**) Comparison of error values; (**b**) Feature contribution change comparison.

**Figure 11:**
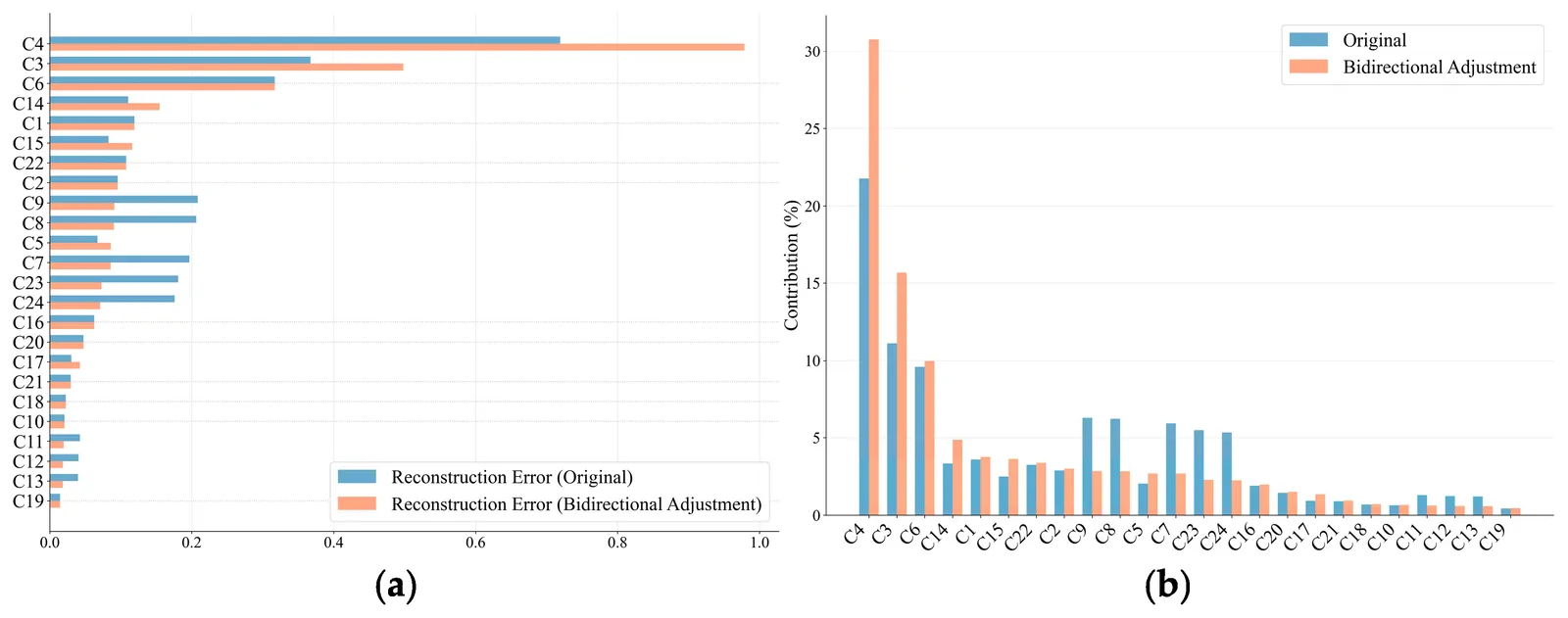
Fault localization results for Case two: (**a**) Comparison of error values; (**b**) Feature contribution change comparison.

A comparison of the error values in Figure 10a and Figure 11a reveals that the gearbox oil temperature (C22) in Case one (abnormal rise in gearbox oil temperature) is not covered by the six physical constraints, so its adjustment factor remains close to 1.0 and its error values are essentially unchanged after bidirectional adjustment, whereas the nacelle temperature (C4) in Case two (abnormal rise in nacelle temperature) is directly governed by the nacelle temperature ordering constraint among the six physical constraints and violates it, so its error values are increased under the penalty mechanism. These findings further substantiate the rationality of the proposed physically-informed bidirectional adjustment of reconstruction errors. Furthermore, the feature contribution change comparisons in Figure 10b and Figure 11b indicate that the proposed method, while achieving fault localization, reshapes the relative contributions of the constrained features, suppresses interference from non-fault features, enhances the contribution ratio of fault features, and improves inter-feature discriminability, thereby enabling high-precision fault localization.

**Figure 12:**
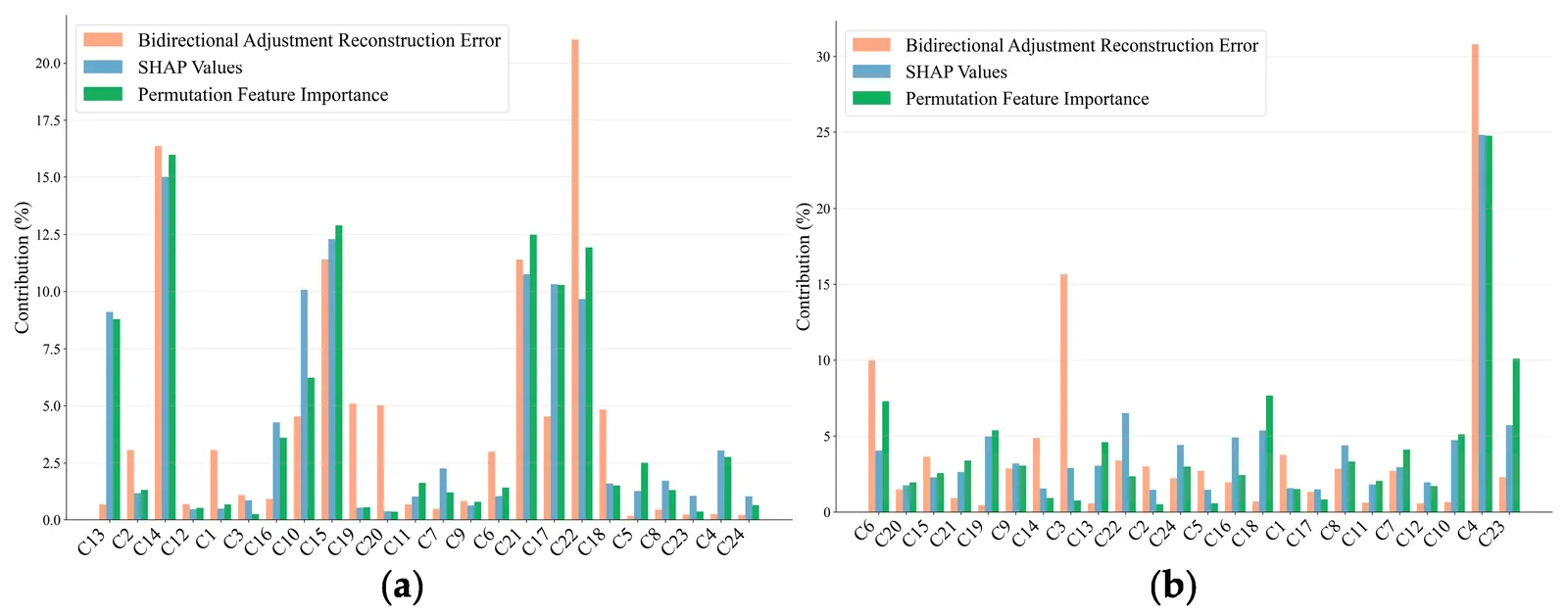
Comparison of fault localization performance among different methods: (**a**) Case one; (**b**) Case two.

Furthermore, the fault localization results obtained by the proposed physically-informed bidirectional adjustment of reconstruction errors are compared with those of the SHAP values method and the Permutation Feature Importance method, as shown in Figure 12. The comparison demonstrates that the proposed method achieves markedly superior fault localization performance over the other two methods.

## 4. Conclusions

A physics-informed framework for SCADA data-based anomaly detection and fault localization is proposed. First, as the validity verification stage of the framework, the embedding validity of explicit algebraic physical constraints is verified based on the autoencoder embedded in MS-DASVDD. Based on two anomaly detection cases from two wind farms in Northern China, the results demonstrate that, even when training samples are insufficient or the training data contain samples violating physical relationships, the introduction of physical information still improves the reconstruction accuracy of normal operational data and achieves the optimal reconstruction performance. In the anomaly detection stage, early anomaly identification is achieved through the MS-DASVDD model, which integrates explicit algebraic physical constraints, latent variable alignment, and transfer learning. The detection threshold is determined by kernel density estimation of the validation-set anomaly scores at a confidence level of 99.7%, which corresponds to the conventional 3*σ* criterion in statistical process control; a confidence-level sensitivity analysis (95.0–99.9%) is further conducted to verify the influence of threshold selection on detection performance, and the results confirm that the 99.7% confidence level lies within a favorable operating region where both false alarms and missed detections are well controlled. The analysis of the above cases further demonstrates that the embedding of explicit algebraic physical relationships, the alignment of latent variables, and the introduction of transfer learning significantly reduce false alarms and missed detections under insufficient training samples, diminish the dependence on training data size, and suppress the interference of data violating physical relationships. The study further investigates the influence of the physical constraint loss weight on the detection efficacy of the proposed model. Although the coupling and trade-off among multiple constraints prevent a single constraint from significantly improving the reconstruction metrics, the model achieves the optimal overall performance through the synergy between multi-physical prior regularization and data-driven fitting, thereby ensuring that each physical constraint takes effect cooperatively. Consequently, the spiky anomaly scores produced by the DASVDD model trained on noisy data violating physical relationships are effectively suppressed on the test set, thereby significantly improving precision, recall, F1 score, and accuracy. On this basis, fault localization is further performed. Finally, in the fault localization stage, a physically-informed bidirectional adjustment reconstruction error method is proposed, which penalizes reconstruction errors consistent with physical constraints while accentuating those inconsistent with them. Grounded in the principle that features violating physical relationships exhibit amplified reconstruction errors during anomalies, this method accurately identifies key variables associated with component faults in wind turbines, substantially enhancing the contribution of fault-related features while markedly reducing that of non-fault features. It outperforms the SHAP values method and the Permutation Feature Importance method in fault localization, providing a solution with significant practical engineering value for wind turbine fault localization.

Despite the valuable findings reported above, the broad applicability of the proposed physics-informed framework still hinges on validation with more complex and more comprehensive datasets. In future work, if more comprehensive and diverse datasets become available, we will further validate and refine the proposed framework to enhance its broad applicability. Meanwhile, the synergistic fusion of graph neural networks with heterogeneous detection algorithms will likewise emerge as an important growth point for future exploration.

## Figures and Tables

**Table 1:** The details of wind turbine SCADA data.

IDX	Name of Variable	Unit	IDX	Name of Variable	Unit
C1	Wind speed	m/s	C14	High-speed shaft bearing temperature	°C
C2	Wind direction	°	C15	Low-speed shaft bearing temperature	°C
C3	Ambient temperature	°C	C16	Generator drive-end bearing temperature	°C
C4	Nacelle temperature	°C	C17	Generator non-drive-end bearing temperature	°C
C5	Cabinet temperature	°C	C18	Generator winding U temperature	°C
C6	Output power	kW	C19	Generator winding V temperature	°C
C7	Pitch motor temperature 1	°C	C20	Generator winding W temperature	°C
C8	Pitch motor temperature 2	°C	C21	Gearbox axis temperature	°C
C9	Pitch motor temperature 3	°C	C22	Gearbox oil temperature	°C
C10	Pitch angle	°	C23	Rotor speed	Rpm
C11	Pitch angle 1	°	C24	Generator speed	Rpm
C12	Pitch angle 2	°	B1	Gearbox cooling water pump status	-
C13	Pitch angle 3	°	B2	Generator cooling fan status	-

**Table 2:** Mathematical expressions of explicit algebraic physical constraints.

Index	Mathematical Expression	Description
①	$\left\{\begin{array}{l} C11-C12=0\\ C12-C13=0 \end{array}\right.$	Equality
②	$\frac{d\left(C1^{3}\right)}{\mathrm{dt}}-k\frac{\mathrm{dC}6}{\mathrm{dt}}=0,\left(k>0\right)$	Correlation
③	$\left\{\begin{array}{l} C7-C8=0\\ C8-C9=0 \end{array}\right.$	Equality
④	$\frac{\mathrm{dC}24}{\mathrm{dt}}-k\frac{\mathrm{dC}23}{\mathrm{dt}}=0,\left(k>0\right)$	Equality
⑤	$\left\{\begin{array}{l} B1=0,C14\leq C15\\ B1=1,C14\geq C15 \end{array}\right.$	Inequality
⑥	$\left\{\begin{array}{l} C18-C19=0\\ C19-C20=0 \end{array}\right.$	Equality
⑦	$\left\{\begin{array}{l} B2=0,\frac{\mathrm{dC}17}{\mathrm{dt}}\geq 0\\ B2=1,\frac{\mathrm{dC}17}{\mathrm{dt}}\leq 0 \end{array}\right.$	Monotonicity
⑧	*C*5 *> C*4 *> C*3	Inequality

**Table 3:** Summary of wind turbine physical constraints.

Index in Table 2	Physical Relationship	Applicable Condition	Tolerance Parameter	Associated Loss
①	Pitch angle constraint	Normal power-generating operation of the unit	*τ_pitch_*	*L_pitch_*
④	Rotor speed ratio constraint	Grid-connected normal power generation of the unit	*τ_speed_*	*L_speed_*
⑧	Nacelle temperature ordering constraint	Normal ventilated cooling of the nacelle	*τ_cba_*	*L_cba_*
③	Pitch motor temperature constraint	Normal steady-state operation inside the hub	*τ_motor_*	*L_motor_*
⑤	Gearbox cooling pump constraint	Normal unit operation, outside the transient window of cooling system switching	*τ*_1_	*L_pump_*
⑦	Generator cooling fan constraint	Normal unit operation, outside the delay window after turbine start-stop	*τ*_2_	*L_fan_*

**Table 4:** Dataset description for Case one.

Dataset	Role	Time Period	Number of Samples Used
Source-domain dataset	Pretraining (80%)	1 to 10 December 2025	11,136
Target-domain dataset	Fine-tuning (85%)	18 to 21 December 2025	4841
Reconstruction-fidelity validation dataset	Validation	13 to 17 December 2025	6575

**Table 5:** Parameter settings of the autoencoder for Case one.

Name of Parameter	Source Domain Settings	Name of Parameter	Target Domain Settings
Autoencoder network	[24, 16, 8, 4, 8, 16, 24]	Autoencoder network	[24, 16, 8, 4, 8, 16, 24]
Batch size	32	Batch size	32
Learning rate	0.001	Learning rate	0.001
Epoch size	10	Epoch size	30
Frozen layers	0	Frozen layers	2
Input dimension	24	Input dimension	24
Latent features (*Zc*)	4	Latent features (*Zc*)	4
Reconstruction error features	2	Reconstruction error features	2
*L*	60	*L*	60
*S*	30	*S*	30
Activation function	tanh	Activation function	tanh
Optimizer	Adam	Optimizer	Adam
Normalization	[0, 1]	Normalization	[0, 1]

**Table 6:** Parameter settings of the six physical constraints for Case one.

Physical Constraint	Name of Parameter	Case One
Pitch angle constraint	*τ_pitch_*	0.05
	*ω_pitch_*	1.0
Rotor speed ratio constraint	*θ*	135
	*τ_speed_*	1.5
	*ω_speed_*	2.8
Nacelle temperature ordering constraint	*τ_cba_*	0.01
	*ω_cba_*	1.8
Pitch motor temperature constraint	*τ_motor_*	1.0
	*ω_motor_*	2.4
Gearbox cooling pump constraint	*τ*_1_	0.05
	*η_on_*	0.97
	*η_off_*	0.97
	*ω_pump_*	1.4
Generator cooling fan constraint	*τ*_2_	0.002
	*N*_min_	12
	*D*	3
	*ω_seg_*	1.0
	*ω_fan_*	3.6

**Table 7:** Evaluation metrics of various models for Case one.

	A	B	C	D	E	F	G	H	I
Respective physics constraint weights	0	1.0	2.4	1.8	2.8	1.4	3.6	-	-
RMSE	36.57	33.54	34.99	33.35	31.14	34.80	34.01	33.42	29.59
MAE	8.30	7.36	7.82	7.50	6.77	8.00	7.57	7.34	6.98
WAPE	4.94	4.38	4.65	4.46	4.03	4.75	4.50	4.36	4.15

**Table 8:** Dataset description for Case two.

Dataset	Role	Time Period	Number of Samples Used
Source-domain dataset	Pretraining (100%)	26 November to 5 December 2025	13,569
Target-domain dataset	Fine-tuning (85%)	11 to 15 December 2025	5374
Reconstruction-fidelity validation dataset	Validation	6 to 10 December 2025	6500

**Table 9:** Parameter settings of the autoencoder for Case two.

Name of Parameter	Source Domain Settings	Name of Parameter	Target Domain Settings
Autoencoder network	[24, 16, 8, 4, 8, 16, 24]	Autoencoder network	[24, 16, 8, 4, 8, 16, 24]
Batch size	32	Batch size	32
Learning rate	0.001	Learning rate	0.001
Epoch size	80	Epoch size	40
Frozen layers	0	Frozen layers	2
Input dimension	24	Input dimension	24
Latent features (*Zc*)	4	Latent features (*Zc*)	4
Reconstruction error features	2	Reconstruction error features	2
*L*	60	*L*	60
*S*	30	*S*	30
Activation function	tanh	Activation function	tanh
Optimizer	Adam	Optimizer	Adam
Normalization	[0, 1]	Normalization	[0, 1]

**Table 10:** Parameter settings of the six physical constraints for Case two.

Physical Constraint	Name of Parameter	Case Two
Pitch angle constraint	*τ_pitch_*	0.05
	*ω_pitch_*	1.0
Rotor speed ratio constraint	*θ*	135
	*τ_speed_*	1.5
	*ω_speed_*	1.0
Nacelle temperature ordering constraint	*τ_cba_*	0.01
	*ω_cba_*	1.0
Pitch motor temperature constraint	*τ_motor_*	1.0
	*ω_motor_*	0.8
Gearbox cooling pump constraint	*τ*_1_	0.05
	*η_on_*	0.71
	*η_off_*	0.71
	*ω_pump_*	0.2
Generator cooling fan constraint	*τ*_2_	0.002
	*N*_min_	20
	*D*	4
	*ω_seg_*	1.0
	*ω_fan_*	1.4

**Table 11:** Evaluation metrics of various models for Case two.

	A	B	C	D	E	F	G	H	I
Respective physics constraint weights	0	1.0	0.8	1.0	1.0	0.2	1.4	-	-
RMSE	52.32	42.54	37.98	51.70	37.72	42.53	43.14	42.64	38.38
MAE	11.31	10.08	8.32	11.00	8.90	9.96	9.16	10.18	9.39
WAPE	7.64	6.81	5.62	7.43	6.01	6.73	6.19	6.88	6.34

**Table 12:** Dataset configurations for the MS-DASVDD anomaly detection model for Case one.

Dataset	Collection Date	Health State	Sample Sizes	Turbine Identity *	Faulty Samples	Purpose
Source-domain dataset (75%)	1 to 10 December 2025	Healthy	10,440	30 MW wind farm	0	Pretraining
Target-domain dataset (85%)	18 to 21 December 2025	Healthy	4841	30 MW wind farm	0	Fine-tuning
Threshold calibration dataset (15%)	18 to 21 December 2025	Healthy	855	30 MW wind farm	0	Threshold determination
Test dataset	22 to 26 December 2025	Containing anomaly	5652	30 MW wind farm	107	Model evaluation

* The original SCADA records do not contain turbine-level ID labels; all data were provided at the wind farm level.

**Table 13:** Training network parameters for Case one.

Name of Parameter	Source Domain Settings	Name of Parameter	Target Domain Settings
Autoencoder network	[24, 16, 8, 4, 8, 16, 24]	Autoencoder network	[24, 16, 8, 4, 8, 16, 24]
Batch size	32	Batch size	32
Learning rate	0.001	Learning rate	0.001
Epoch size 1	40	Epoch size 2	30
*ν*	0.2	*ν*	0.2
*γ*	3	*γ*	3
*λ*_1_	1.6	*λ*_1_	1.6
*λ*_2_	1.2	*λ*_2_	1.2
*α*	18	*α*	18
*L*	60	*L*	60
*S*	30	*S*	30
Early-stopping patience	-	Early-stopping patience	5
Frozen layers	0	Frozen layers	1
Input dimension	24	Input dimension	24
Latent features (*Zc* + *Zphy*)	8	Latent features (*Zc* + *Zphy*)	8
Reconstruction error features	2	Reconstruction error features	2
SVDD input dimension	10	SVDD input dimension	10
Activation function	tanh	Activation function	tanh
Optimizer	Adam	Optimizer	Adam
Normalization	[0, 1]	Normalization	[0, 1]

**Table 14:** Anomaly detection results of MS-DASVDD under different confidence levels for Case one.

Confidence Level	Threshold	Precision	Recall	F1 Score	Accuracy
95.0%	0.009	0.2709	1.0000	0.4263	0.9490
99.0%	0.017	0.5047	1.0000	0.6708	0.9814
99.5%	0.024	0.6731	0.9813	0.7985	0.9906
99.7%	0.031	0.8696	0.9346	0.9009	0.9961
99.9%	0.042	0.9880	0.7664	0.8632	0.9954

**Table 15:** Comparative results for Case one.

Method	Precision	Recall	F1 Score	Accuracy
DASVDD	0.5873	0.3458	0.4353	0.9830
DASVDD with Physical Constraints	0.2990	0.5421	0.3854	0.9673
DASVDD with Physical Constraints and Latent Alignment	0.6372	0.6729	0.6545	0.9866
MS-DASVDD	0.8696	0.9346	0.9009	0.9961

**Table 16:** Dataset configurations for the MS-DASVDD anomaly detection model for Case two.

Dataset	Collection Date	Health State	Sample Sizes	Turbine Identity *	Faulty Samples	Purpose
Source-domain dataset (80%)	26 November to 5 December 2025	Healthy	10,855	40 MW wind farm	0	Pretraining
Target-domain dataset (85%)	11 to 15 December 2025	Healthy	5374	40 MW wind farm	0	Fine-tuning
Threshold calibration dataset (15%)	11 to 15 December 2025	Healthy	949	40 MW wind farm	0	Threshold determination
Test dataset	16 to 18 December 2025	Containing anomaly	2860	40 MW wind farm	114	Model evaluation

* The original SCADA records do not contain turbine-level ID labels; all data were provided at the wind farm level.

**Table 17:** Training network parameters for Case two.

Name of Parameter	Source Domain Settings	Name of Parameter	Target Domain Settings
Autoencoder network	[24, 16, 8, 4, 8, 16, 24]	Autoencoder network	[24, 16, 8, 4, 8, 16, 24]
Batch size	32	Batch size	32
Learning rate	0.001	Learning rate	0.001
Epoch size 1	40	Epoch size 2	40
*ν*	0.1	*ν*	0.1
*γ*	4	*γ*	4
*λ*_1_	0.8	*λ*_1_	0.8
*λ*_2_	1.8	*λ*_2_	1.8
*α*	10	*α*	10
*L*	60	*L*	60
*S*	30	*S*	30
Early-stopping patience	-	Early-stopping patience	5
Frozen layers	0	Frozen layers	2
Input dimension	24	Input dimension	24
Latent features (*Zc* + *Zphy*)	8	Latent features (*Zc* + *Zphy*)	8
Reconstruction error features	2	Reconstruction error features	2
SVDD input dimension	10	SVDD input dimension	10
Activation function	tanh	Activation function	tanh
Optimizer	Adam	Optimizer	Adam
Normalization	[0, 1]	Normalization	[0, 1]

**Table 18:** Anomaly detection results of MS-DASVDD under different confidence levels for Case two.

Confidence Level	Threshold	Precision	Recall	F1 Score	Accuracy
95.0%	0.004	0.4351	1.0000	0.6064	0.9483
99.0%	0.013	0.9217	0.9298	0.9258	0.9941
99.5%	0.019	0.9712	0.8860	0.9266	0.9944
99.7%	0.025	1.0000	0.7544	0.8600	0.9902
99.9%	0.032	1.0000	0.6754	0.8063	0.9871

**Table 19:** Comparative results for Case two.

Method	Precision	Recall	F1 score	Accuracy
DASVDD	0.4235	0.6316	0.5070	0.9510
DASVDD with Physical Constraints	0.3532	0.6228	0.4508	0.9395
DASVDD with Physical Constraints and Latent Alignment	0.6283	0.6228	0.6256	0.9703
MS-DASVDD	1.0000	0.7544	0.8600	0.9902

**Table 20:** Fault localization parameter settings for Case one and Case two.

Name of Parameter	Case One	Case Two
*ξ*	0.85	0.60
*ρ*	0.15	0.40
*ο*	0.80	0.30
*ψ*	20	20
*a*_min_	0.1	0.1
*a*_max_	2.0	2.0
*L*	60	60
*S*	30	30

## Data Availability

The data presented in this study are available on request from the corresponding author due to national policies and industry confidentiality.
